# Highly Thermally Conductive and Structurally Ultra-Stable Graphitic Films with Seamless Heterointerfaces for Extreme Thermal Management

**DOI:** 10.1007/s40820-023-01277-1

**Published:** 2023-12-19

**Authors:** Peijuan Zhang, Yuanyuan Hao, Hang Shi, Jiahao Lu, Yingjun Liu, Xin Ming, Ya Wang, Wenzhang Fang, Yuxing Xia, Yance Chen, Peng Li, Ziqiu Wang, Qingyun Su, Weidong Lv, Ji Zhou, Ying Zhang, Haiwen Lai, Weiwei Gao, Zhen Xu, Chao Gao

**Affiliations:** 1 MOE Key Laboratory of Macromolecular Synthesis and Functionalization, Department of Polymer Science and Engineering, Key Laboratory of Adsorption and Separation Materials and Technologies of Zhejiang Province, Zhejiang University, 38 Zheda Road, Hangzhou, 310027 People’s Republic of China; 2 Shanxi-Zheda Institute of Advanced Materials and Chemical Engineering, Taiyuan, 030032 People’s Republic of China; 3 Beijing Spacecrafts Manufacturing Co., Ltd, Beijing Friendship Road 104, Haidian District, Beijing, 100094 People’s Republic of China; 4 Beijing Institute of Space Mechanics and Electricity, Beijing Friendship Road 104, Haidian District, Beijing, 100094 People’s Republic of China; 5 China Academy of Aerospace Aerodynamics, Beijing, 100074 People’s Republic of China; 6 Hangzhou Gaoxi Technol Co., Ltd, Hangzhou, 311113 People’s Republic of China; 7 International Research Center for X Polymers, International Campus, Zhejiang University, Haining, 314400 People’s Republic of China

**Keywords:** Highly thermally conductive, Structurally ultra-stable, Graphitic film, Extreme thermal management, Liquid nitrogen bubbling

## Abstract

Highly thermally conductive graphitic film (GF) materials have become a competitive solution for the thermal management of high-power electronic devices. However, their catastrophic structural failure under extreme alternating thermal/cold shock poses a significant challenge to reliability and safety. Here, we present the first investigation into the structural failure mechanism of GF during cyclic liquid nitrogen shocks (LNS), which reveals a bubbling process characterized by “permeation-diffusion-deformation” phenomenon. To overcome this long-standing structural weakness, a novel metal-nanoarmor strategy is proposed to construct a Cu-modified graphitic film (GF@Cu) with seamless heterointerface. This well-designed interface ensures superior structural stability for GF@Cu after hundreds of LNS cycles from 77 to 300 K. Moreover, GF@Cu maintains high thermal conductivity up to 1088 W m^−1^ K^−1^ with degradation of less than 5% even after 150 LNS cycles, superior to that of pure GF (50% degradation). Our work not only offers an opportunity to improve the robustness of graphitic films by the rational structural design but also facilitates the applications of thermally conductive carbon-based materials for future extreme thermal management in complex aerospace electronics.

**Supplementary Information:**

The online version contains supplementary material available at 10.1007/s40820-023-01277-1.

## Highlights

- Presenting the first investigation into the structurally bubbling-failure mechanism of graphitic film during cyclic liquid nitrogen shocks.
- Proposing an innovative design about seamless heterointerface constructing a Cu-modified structure.
- Inventing a new ultra-stable species of highly thermally conductive films to inspire new techniques for efficient and extreme thermal management.

**Supplementary Information:**

The online version contains supplementary material available at 10.1007/s40820-023-01277-1.

## Graphical abstract

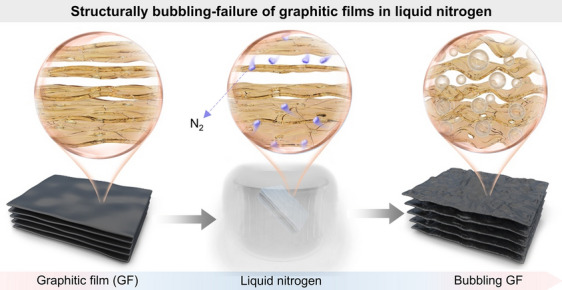

## Introduction

The continuing miniaturization and integration of ultra-fast high-frequency electronic devices have led to significant challenges in efficient thermal management, as it inevitably increases temperature and reduces reliability [1–3]. These challenges are particularly apparent in complex operating conditions, such as militaries [4], spacecrafts [5], supercomputers [6], nuclear reactors [7–9], and other complex scenarios. Some common environmental factors in these extreme application scenarios like temperature variations [10, 11], ultraviolet irradiation [12], atomic oxygen [12], and liquid nitrogen [13–15] can significantly impact the stability of devices, setting new requirements for thermal management materials.

Liquid nitrogen, with its incredibly low temperature of 77 K, is a versatile cold source used in nuclear power plants and aerospace [15–17]. Heat dissipation includes the process of heat production from devices, heat transfer (heat conduction) by thermal management materials, and cooling by cold source. In these special working scenarios, mass waste heat generated by high-power devices (heat source) could be conducted into liquid nitrogen (cold source) through a heat transfer medium [16, 17]. However, this technology faces challenges due to the exceptionally low temperature, high surface tension, and significant volume expansion under temperature changes of liquid nitrogen [18]. These factors can lead to unavoidable structural damage of functional parts under such complicated service conditions, thereby degrading heat dissipation performance. Therefore, achieving the integration of structural stability and high-performance material design under extreme conditions is a critical challenge in the field of thermal management.

**Fig. 1:**
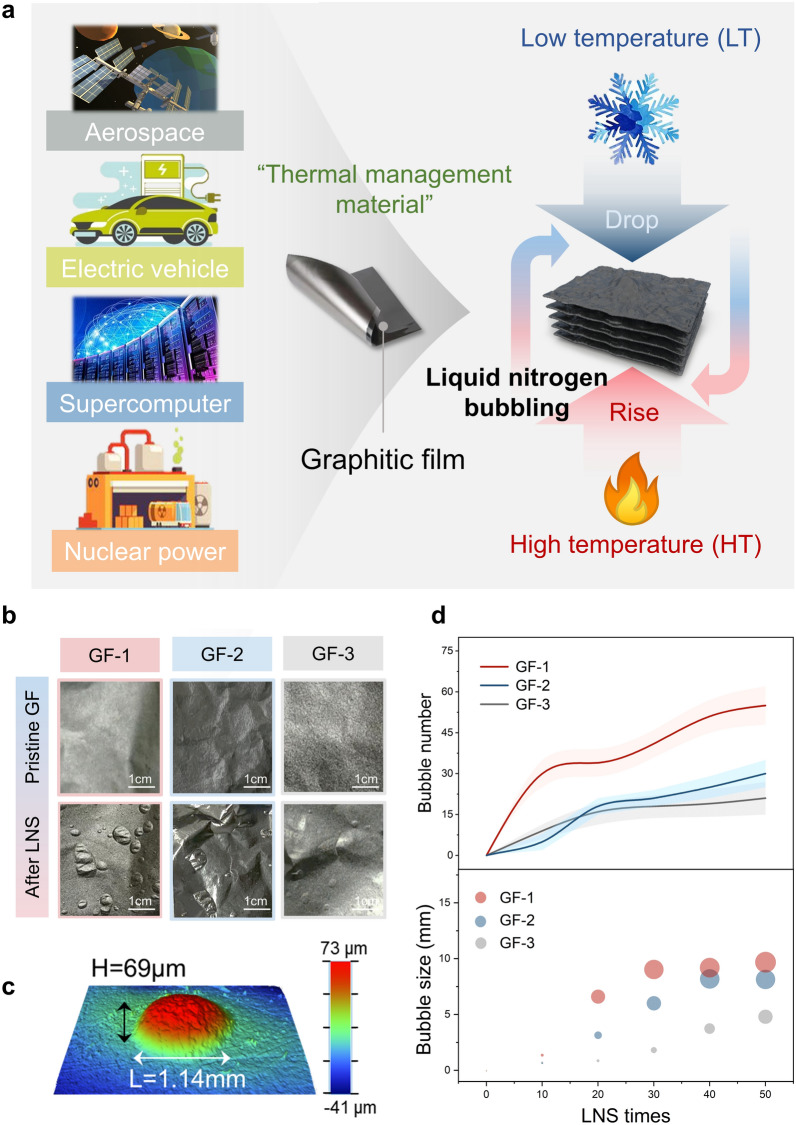
Structurally bubbling-failure mechanism of GF during extreme environment. **a** Schematic illustration of GF applied in temperature fluctuation scenarios. **b** Morphologies of different GFs before and after LNS. **c** Stereoscopic structure image of a typical bubble. **d** The number and size of bubbles on the surface of GFs vary with the number of LNS

Thermal management materials including polymers [19–21], ceramics [22–29], and metals [30–32] have been extensively utilized and developed. Due to their inherent drawbacks, they are unable to meet the demand for complex and extreme scenarios. Carbon-based materials [33–44], such as highly thermally conductive graphitic film (GF) with the combined merits of low density, outstanding flexibility, low thermal expansion coefficient, and intrinsic chemical resistance offer a promising alternative to traditional thermal conductive materials [45–48]. At present, there are two main ways to achieve high structure stability and performance at room temperature, including the interlayer crosslinking strategy [49, 50] and the plasticizing orientation method [51, 52]. However, utilizing cross-linked polymers would reduce the thermal conductivity, and plasticization stretching could only eliminate the partial internal defects of films, but its intrinsic structural instability could not be solved under extreme conditions. Besides, these methods are mainly concerned with film properties at room temperature. There are few studies on the structural stability and durability of GF in extreme scenarios (Fig. 1a), such as liquid nitrogen cyclic thermal management in nuclear power plants [15] and compute overclocking operation [16, 53]; extremely low or high temperatures from − 180 to + 180 °C in space; low-temperature service scene of electric vehicle in the cold regions. We wonder if GF can endure the cyclic liquid nitrogen shock (LNS) without sacrificing its thermal properties to achieve long-term stability.

Here, we report the unusual bubbling phenomenon of GF during LNS for the first time and uncover the structural failure mechanism. The bubbling process is related to the permeation and diffusion of N_2_ molecules in the crevices and inner voids of GF. To overcome this issue, we further propose a novel metal-nanoarmor strategy involving sputtering copper coatings on both surfaces of GF (GF@Cu). This well-designed seamless heterogeneous structure provides superior structural stability of GF@Cu after hundreds of LNS from 77 to 300 K. The GF@Cu exhibits a high thermal conductivity up to 1088 W m^−1^ K^−1^, with a retention ratio higher than 95%, which greatly exceeds that of pure GF (~ 50% degradation). Furthermore, the seamless interface design also ensures improved mechanics, electricity, and magnetism properties compared to the original GF. This study overcomes the intrinsic structural instability of highly thermally conductive GF in extreme liquid nitrogen oscillation environments. Additionally, the revealed failure mechanism of cyclic LNS and the metal-nanoarmor strategy with seamless interface provide inspiration for the development of extreme thermal management materials and technologies.

## Experimental Section

### Materials

Highly thermally conductive graphitic films were purchased from Hangzhou Gaoxi Technology Co., Ltd (www.gaoxitech.com). All chemicals were analytically pure, all materials were used as received.

### Preparation of Cu-modified Graphitic Film (GF@Cu)

Cu coating (~ 240 nm) has been deposited by magnetron sputtering at an Ar partial pressure of 4.5 × 10^−2^ mbar, with a vacuum of 10^−5^ mbar, with a cathode power of 120 W onto graphitic film substrates for 30 min. The graphitic films were already cleaned with ethanol.

### Characterizations

The instrument used for magnetron sputtering was Kurt J. Lesker PVD 75. Stereoscopic structure images were taken by the white-light interferometer (Wyko NT9100). The microstructure and mapping of GF@Cu were characterized by a field-emission scanning electron microscope (SEM) with energy dispersive X-ray (Hitachi S4800). Internal structure images were obtained by a focused ion beam and imaged on the same instrument (Carl Zeiss Auriga 40). X-ray diffraction (XRD) was performed with an X’Pert Pro (PANalytical) diffractometer using monochromatic Cu Kα1 radiation (*λ* = 1.5406 Å) at 40 kV. X-ray photoelectron spectroscopy (XPS) characterization was conducted using a ThermoFisher Escalab 250Xi instrument and all binding energies were referenced to the C 1*s* neutral carbon peak at 284.8 eV. Raman spectroscopy and mapping scanning were recorded by a commercial Renishaw in a Via-Reflex Raman microscope at an excitation wavelength of 532 nm. Nanoindentation experiments were conducted at a constant temperature of 20 °C on Agilent Nano Indenter G200 with a standard Berkovich indenter. The thermal conductivity was evaluated by NETZSCH LFA467 HyperFlash at room temperature, which could obtain a relatively effective value of in-plane thermal conductivity of the thin film with a similar layered structure. The interfacial microstructure of GF@Cu was observed by scanning transmission electron microscope (Titan ChemiSTEM). Transmission electron microscope samples were prepared by a focused ion beam (FIB) machine (Nova 600, FEI). Tensile strength tests were conducted using an Instron 2344 at a loading rate of 5 mm min^−1^, and the size of the tested strip is 20 mm (length) × 1 mm (width). The gauge length is 10 mm. The bend speed was about six cycles per minute in the bending test. The electrical conductivity and resistance were measured using a standard four-probe method on a Keithley 2460 multiple-function source meter. An infrared camera (FLIR T630sc) was used to measure the temperature profile along with the film strips. The EMI SE performance was tested by a vector network analyzer (ZNB-40, Rohde & Schwarz, Germany).

## Results and Discussion

### Environmental Tolerance and Bubbling Failure of GF

To investigate the tolerance of GF in extreme environments, specifically rapid shocks from air to liquid nitrogen, a cyclic LNS test is introduced. The GFs were repeatedly subjected to LNS to assess their structural robustness (Fig. 1a). All three GFs exhibit an obvious bubbling phenomenon and the number of bubbles increases with LNS times, indicating serious structural damage (Figs. 1b and S1). Similarly, this bubbling phenomenon is also observed in the graphene film (Fig. S2), indicating that it may be a common phenomenon in carbon-based films. A typical morphology of a bubble with a length of 1.14 mm and a height of 69 μm is illustrated in Fig. 1c (additional optical profiler images of bubbles are provided in Fig. S3). The bubble evolution processes in all GFs are evaluated with LNS times, showing a significant positive correlation between the LNS times and the number/size of bubbles on the surface of GF (Fig. 1d). This bubbling process follows the classical nucleation-growth model [54, 55]. Furthermore, it is observed that GF with higher density tends to exhibit fewer numbers and smaller size bubbles per unit area (Fig. S4), indicating a relationship between the compactness structure of GF and the bubbling degree. Among them, when GF-1 with a density of 1.84 g cm^−3^ suffered from 50 times LNS, the number of bubbles reached an astonishing 55, and its bubble size was up to 9.68 mm.

### Structural Failure Mechanism and Molecular Dynamics Simulation of Bubbling Process in GF

**Fig. 2:**
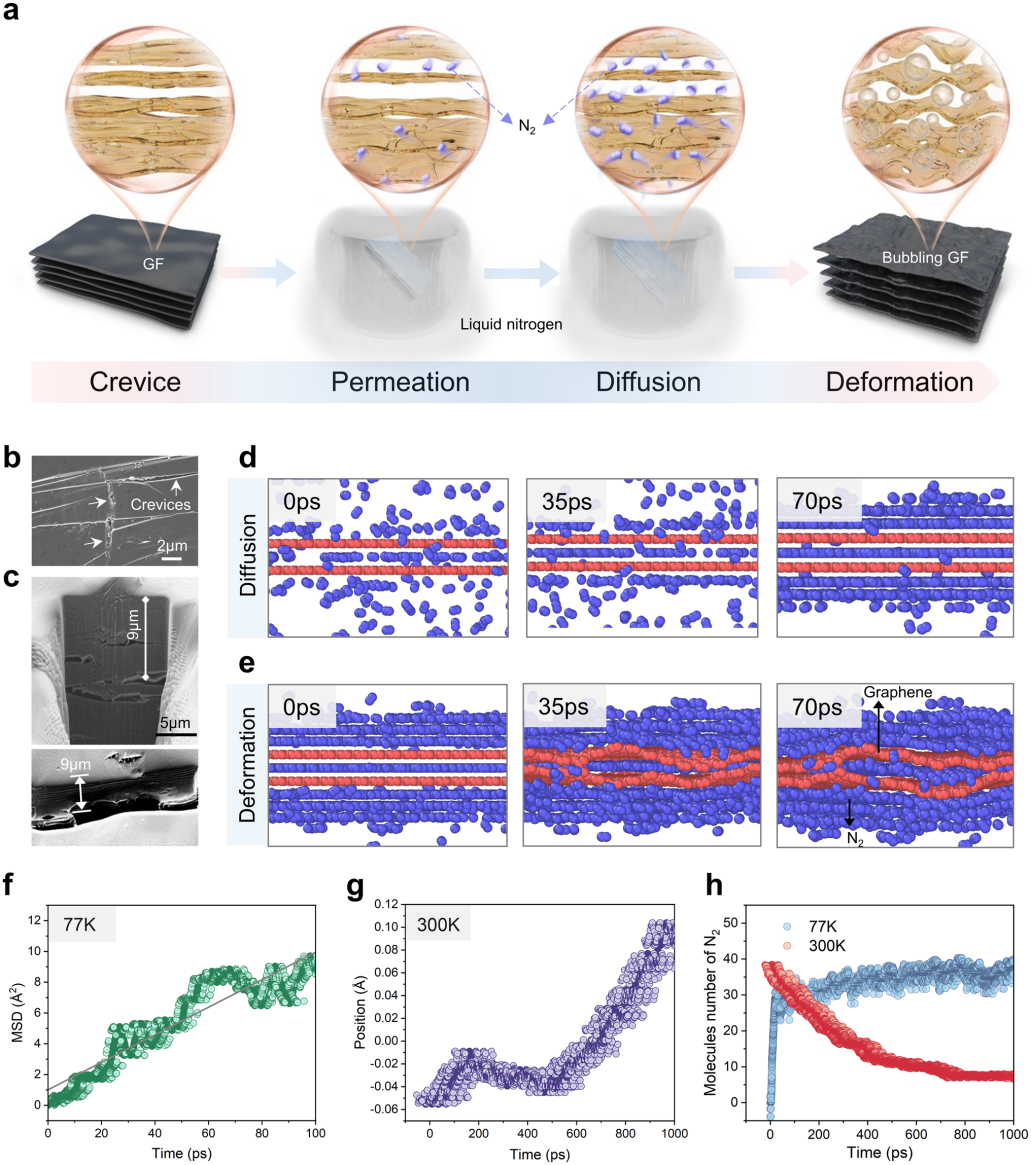
Structural failure mechanism of GF during cyclic LNS. **a** Schematic diagram of GF deformation caused by N_2_ molecules. **b** SEM image of the crevices on GF. **c** SEM images of the internal structure of GF and the bubble wall, obtained by using a focused ion beam cutting. **d** Corresponding 3D rendering of N_2_ molecules diffuse over time at liquid nitrogen temperature (77 K). **e** Corresponding 3D rendering of N_2_ molecules diffuse over time as the ambient temperature becomes room temperature (300 K). **f** The mean square displacements (MSD) of N_2_ molecules from MD simulations at 77 K. **g** The average position of the graphene sheet in a nitrogen environment from MD simulations at 300 K. The position in center of two graphene sheets slit is defined as 0. **h** Molecules number of N_2_ between graphene sheets over time from MD simulations at different temperatures

To uncover the possible mechanism of bubbling process during LNS, the GF immersed directly in liquid nitrogen was compared with the GF experienced LNS without contact with liquid nitrogen by plastic sealing. The latter shows no bubbles on the surface (Fig. S5). Hence, these results suggest that the interaction between liquid nitrogen molecules and GF is a key factor in the bubble formation process. A structural failure mechanism is proposed to elucidate the bubbling process of GF during LNS, as depicted in Fig. 2a. When GF is immersed in liquid nitrogen, N_2_ molecules permeate through the surface crevices (Fig. 2b) and accumulate within the inner voids of the GF due to the ultralow critical diffusion resistance [56, 57]. Upon moving the GF into the air, the sudden temperature change triggers the transition of the N_2_ permeated in the GF from the liquid phase to the gas phase, leading to huge volume expansion (~ 648 times expansion in unconfined space) and local layer deformation. This deformation ultimately results in the formation of bubbles, which is also consistent with our observations in the practical LNS tests (see Video S1), where the surface bubbles fast expand as the liquid nitrogen evaporates. To further support our proposed mechanism, the cross-sections of GF were observed through the FIB-SEM system (Fig. 2c). There are voids between the layers of GF, which are related to the assembly of precursor in fabrication process and the escape of volatile components during post-processing stage [58–60]. Furthermore, comparable internal structures are also observed in several GFs that exhibit bubbling during LNS (Fig. S6). The consistent thickness observed in the bubble walls (~ 9 μm) and the void depth (~ 9 μm) also provides evidence for this structural failure mechanism (Fig. 2c).

To further verify the mechanism that the crevices and inner voids in GF facilitate the diffusion of N_2_ and subsequent bubble formation, we conducted molecular dynamics simulations to explore the interaction between N_2_ and graphene sheets under alternating temperature conditions (further details can be found in the Supporting Information). It is evident that N_2_ molecules exhibit continued diffusion at a temperature of 77 K with low drag, resulting in a gradual accumulation of N_2_ molecules in the interlayer spaces of graphene (Fig. 2d, h). N_2_ molecules diffusion process can also be illustrated from the mean square displacements (MSD) curve (Fig. 2f), whose slope contains information about the degree of diffusion. The diffusion coefficient (1.48 × 10^–8^ m^2^ s^−1^) for N_2_ molecules is calculated from their mean square displacements, indicating that N_2_ molecules can diffuse into the graphene sheets. Upon increasing the temperature to 300 K, the graphene sheets show significant deformation of ~ 0.12 Å in the perpendicular direction to the surface, which is consistent with the observed bubbling process (Figs. 2e, g and S7). Therefore, the structural failure behavior of the GF is strongly related to the surface and internal defects, which provides theoretical guidance for improving the structural stability of GF during cyclic LNS.

### Construction and Characterization of Seamless Heterointerface

**Fig. 3:**
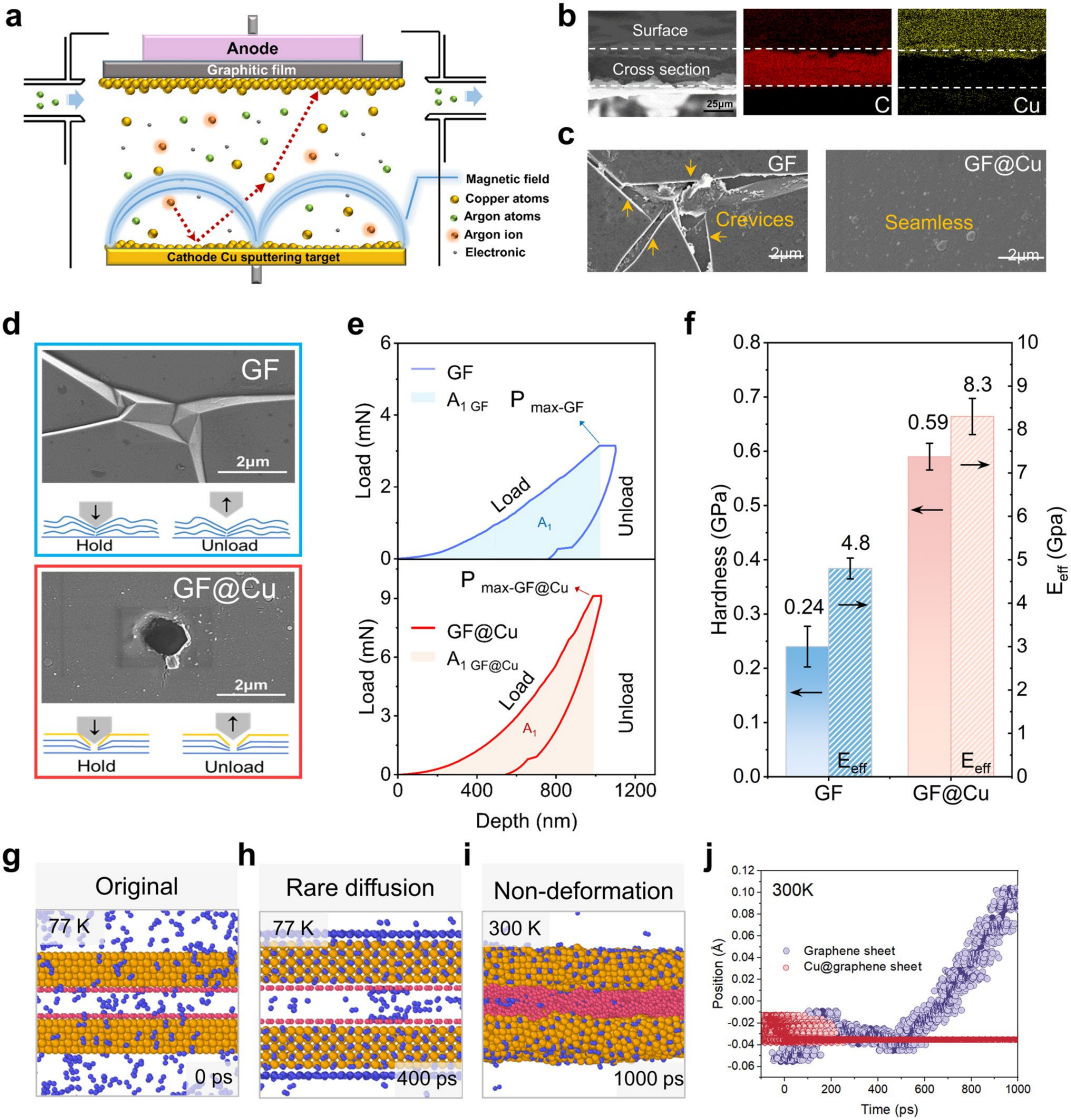
Design of seamless heterointerface constructing a Cu-modified structure. **a** Schematic diagram of magnetron sputtering coating with Cu on the surface of GF. **b** Cross-sectional energy dispersive X-ray (EDX) mapping of GF@Cu. **c** SEM images of the surface of GF and GF@Cu. **d** Morphology inspection of indentation on GF and GF@Cu surface after nanoindentation test. **e** Force versus depth curves of GF and GF@Cu by nanoindentation. **f** Hardness and effective Young’s modulus of GF and GF@Cu. **g** Corresponding 3D rendering of Cu@graphene silt under liquid nitrogen environment (77 K). **h** Corresponding 3D rendering of rare N_2_ molecules diffuse into Cu@graphene silt over time at liquid nitrogen temperature (77 K). **i** Corresponding 3D rendering of Cu@graphene sheets hardly deform as the ambient temperature becomes room temperature (300 K). **j** The average position of the graphene sheet and Cu@graphene sheet in a nitrogen environment from MD simulations at 300 K. The position in center of two graphene sheets slit is defined as 0

To enhance the structural stability of GF and ensure their high performance in extreme environments, it is crucial to mitigate the defect constraint on the surface. A novel metal-nanoarmor strategy is proposed to construct a seamless heterogeneous interface on the surface of GF by using magnetron sputtering ultra-thin Cu layer (Fig. 3a), which could suppress bubbles by restrain the bubble nucleation and growth from Henry theory [54]. The GF@Cu possesses a compact and uniform Cu layer with ~ 240 nm (Figs. 3b and S8, S9), showing characteristic peak of Cu element in XPS spectra (Fig. S10). From the density functional theory (DFT) calculation, the interfacial strength at Cu/graphene interface of 0.516 J m^−2^ is significantly higher than that of the graphene/graphene interface (0.259 J m^−2^), which is attributed to the strong interaction of Cu 3*d* and C 2*p* states. The charge transfer between interfacial atoms leads to strong interfacial strength (Fig. S11). Moreover, the GF@Cu with seamless interface retains the same graphitization degree as the original GF, indicating that the crystal structure of graphite layer is not significantly damaged (Fig. S12). The ultra-thin and seamless heterogeneous interface structure of GF@Cu could effectively prevent the diffusion of N_2_ molecules via defect on the surface (Fig. 3c), resulted from the defect density decreased from ~ 9.6% to ~ 0% compared to the original GF (Figs. S13 and S15). This enhanced seamless heterointerface can effectively restrain the diffusion of N_2_ molecules and then reduce the bubble nucleation (Fig. S13). The molecular dynamics results also show that the addition of nano-metal armor effectively reduces the accumulation of N_2_ in the interior and thus could decrease the probability of nucleation to avoid catastrophic bubbling (Fig. 3g, h). Meanwhile, the seamless heterointerface could effectively inhibit bubble growth through enhanced surface mechanical properties. Nano-indentation micromechanical characterization was employed to explore the surface deformability of both the GF and the GF@Cu to elucidate the enhancement mechanism for seamless heterogeneous interface. A permanent inverted hole is generated on the surface of the GF and the GF@Cu by the Berkovich indenter (Figs. 3d and S16). It is evident that the GF without seamless interface has undergone greater deformation as measured by the area of the hole (Table S1). Additionally, the load-depth curve shows that more energy is required to conquer the same depth in the GF@Cu with seamless interface (A_1 GF@Cu_ > A_1 GF_) (Fig. 3e).

The hardness value of GF@Cu is determined to be 0.59 GPa, which guarantees less deformation, while the hardness of GF is only 0.24 GPa (Fig. 3f). Similarly, the effective Young’s modulus (E_eff_) of both films also reflect that the seamless interface could enhance the resistance to deformation under a given stress. The E_eff GF@Cu_ (8.3 GPa) is significantly higher than E_eff GF_ (4.8 GPa) (Fig. 3f). The superior hardness and E_eff_ of GF@Cu with seamless interface confirm its advantages in enhancing deformation resistance, which is essential for reducing complex mechanical damage caused by extreme liquid nitrogen expansion during LNS. This mechanical enhancement would lead to a significant increment in the surrounding pressure of bubble which should be overcome in bubble growth (Fig. S14), and further inhibit the growth of bubbles (Fig. S13). The effectiveness of our seamless heterointerface is also verified by molecular dynamics simulations. The lamellar deformation caused by liquid nitrogen gasification of the model with copper atomic layer is significantly smaller than that of pure graphene film **(**Fig. 3i, j). Additionally, the penetration of N_2_ during LNS is significantly hindered by the protection of metal-nano armor, which would weaken the ability of the bubble growth endowed by the concentration of N_2_ (Figs. S13, S14). The metal-nano armor strategy guidance with seamless interface shows great promise for enhancing the structural stability of conventional GF in extreme environments to conduct thermal management.

### Effects of Seamless Heterointerface on the Thermal Conductivity of GF

**Fig. 4:**
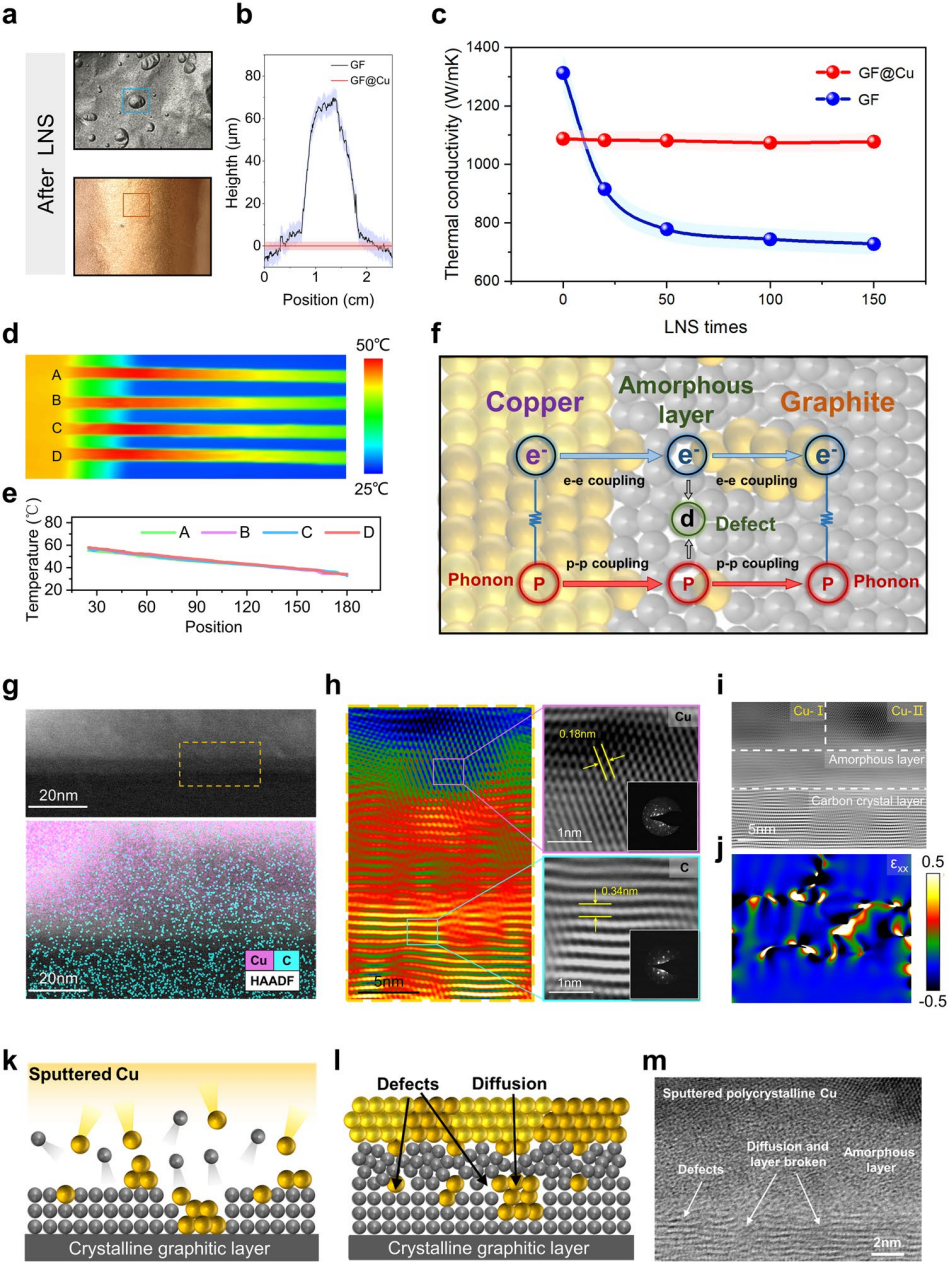
Effects of seamless heterointerface on the thermal conductivity of GF. **a** Digital images and **b** surface information of GF and GF@Cu after 150 times LNS. **c** Thermal conductivity of GF and GF@Cu varies with the number of LNS times. **d** Infrared image of GF@Cu and **e** temperature profiles with different LNS times, A = 0 times, B = 50 times, C = 100 times, D = 150 times. **f** Schematic diagram about heat transport mechanisms at Cu/C interface. The possible heat transport pathways at interface include: the phonon coupling between C and Cu, the electron coupling between C and Cu and interface defect. **g** Element distribution of the Cu/C structure, the HAADF mapping shows interfacial mixing layer forms at the C/Cu interface. **h–i** AC-STEM images of the Cu/C interface. **j** Analysis on local strain of Cu/C interface. **k-l** Cross-sectional schematics and **m** TEM image of magnetron-sputtering-deposited Cu on the surface of GF, where the bombardment of the GF surface by high-energy Cu atoms and clusters creates considerable damage to the GF surface, producing an amorphous layer with apparent defects, interface diffusion, atomic disorder and interfacial stress

The seamless heterointerface has been proven to play an important role in maintaining the structural stability of GF for achieving stable performance. We comprehensively investigated the effect of the seamless heterogeneous interface on the thermal transport of the GF after LNS. When exposed to an equivalent 150 times LNS, the GF@Cu presents a distinctly smooth surface compared with the bubbling surface of GF (Figs. 1b and 4a, b), attributed to the impervious seamless heterointerface. Besides, the interface connection of Cu@GF between the seamless heterointerfaces and the GF substrate is still tight after cyclic LNS (Fig. S17). As a consequence, the interior structure of GF@Cu remains non-penetration, thereby avoiding extensive structural destruction by LNS. As shown in Fig. 4c, the thermal conductivity of the GF drastically decreases by almost 50% from 1312 to 728 W m^−1^ K^−1^ after 150 times LNS. In contrast, the GF@Cu displays higher thermal conductivity retention up to 96%, changing from 1137 to 1088 W m^−1^ K^−1^ after the same time LNS. The corresponding infrared image of the GF@Cu with seamless heterointerface also demonstrates stable thermal transfer performance after LNS, while the structural failure of GF seriously disturbs its heat transfer (Figs. 4d, e and S18). Furthermore, it is worth mentioning that the thermal conductivity of the GF@Cu is slightly lower than that of the theoretical value, according to the classical composite parallel model (Fig. S19). The finite element simulation suggests the interface plays a non-negligible role in this weak reduction of thermal conductivity of GF@Cu with seamless interface before LNS (Fig. S20). When the GF is sputtered with seamless heterogeneous interface, the phonon coupling between Cu and C dominates the interfacial heat transport, and the electron coupling between Cu and C provides an additional heat transport pathway along the C/Cu interface [59]. However, the high-energy magnetron-sputtering-deposited method would generate the inevitable interfacial defects of substrate materials, and the additional defects would impede the interfacial heat transport, thus leading to decreased thermal conductivity [60–63] (Fig. 4f).

To explore this interfacial structure evolution and reveal the atomic origins of the reduction of thermal conductivity, we observed the heterointerface by atomically resolved Aberration Corrected Scanning Transmission Electron Microscopy (AC-STEM). As shown in Fig. 4g, there is an obvious penetration of C and Cu elements at the interface while in the corresponding bulk parts they are homogeneously distributed. According to the AC-STEM (Fig. 4h), the bulk of GF has a typical layered graphene morphology with a layer spacing of 0.34 nm, indicating a high crystallinity to ensure ultra-high thermal conductivity. Meanwhile, the bulk Cu layer at the interface shows a polycrystalline structure, ensuring the sealing function of seamless heterogeneous interface. It is clear that an amorphous layer (a-L) of ∼5 nm exists at the C/Cu interface, caused by high-energy atom bombardment during magnetron sputtering (Fig. 4i). Therefore, the total thermal resistance (R_total_) of the C/Cu interface actually could be considered as the sum of the thermal resistance of a-L (R_a-L_) and the interface itself (R_C/a-L_ + R_a-L/Cu_). The increase of defect density within the a-L could lead to the increment of the corresponding R_a-L_ [64]. Simultaneously, the mismatch of electron density and vibrational density of states at the GF/Cu interface also leads the transport of electrons and phonons to be blocked, resulting in high interfacial thermal resistance at nanoscale [65]. Furthermore, the drastic interface local strain accompanied by high density of defects in the C/Cu interface is inferred to reduce phonon scattering relaxation time and increase phonon thermal resistance [64] (Fig. 4j). The bombardment of high-energy Cu atoms during magnetron sputtering preparation is responsible for inevitably generating apparent defects, interface diffusion, atomic disorder and interfacial stress [66], which are obviously characterized in the TEM image of C/Cu interface (Fig. 4k–m). The appearance of Raman D peak on the surface of the GF@Cu after removing Cu layers also confirms the introduction of defects in the magnetron sputtering process (Fig. S21). Therefore, the existence of R_total_ in the nanoscale C/Cu interface would slightly reduce the thermal conductivity of GF@Cu. It is worth mentioning that this ultra-thin seamless heterointerface can greatly improve the structural stability to maintain the high thermal conductivity of GF after numerous LNS times, so as to meet the needs of extreme thermal management in the future.

### Comprehensive Functional Performance of GF@Cu for Practical Applications

**Fig. 5:**
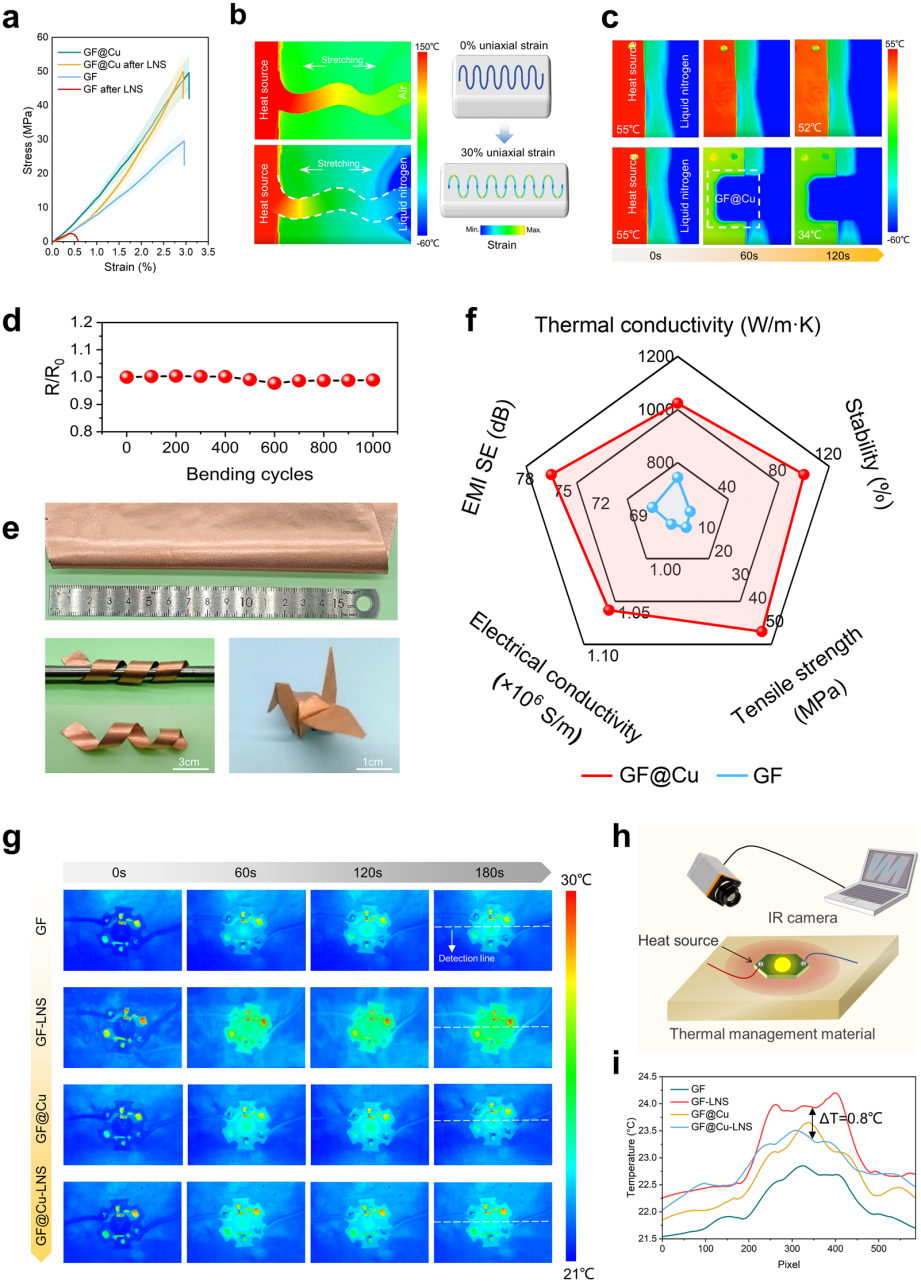
Comprehensive functional performance of GF@Cu for practical applications. **a** Tensile stress–strain curves of GF and GF@Cu. **b** Infrared images and schematic diagrams of GF@Cu as a stretchable and stable thermal management module in extreme environment from 77 to 300 K. **c** Infrared images of heat dissipation application conducted by GF@Cu with liquid nitrogen as cold source. **d** Relative resistance variation of GF@Cu under cyclic bending 1000 times. **e** GF@Cu with the state of bending, twisting, and folding into a crane without breakages. **f** Overall performances of GF@Cu (red line) and GF (blue line). Stability is defined as 100% when no structural damage occurs for ≥ 100 LNS, and stability is defined as the number of shocks when bubbles occur for < 100 LNS. **g** Infrared images of an LED lamp with different films for heat dissipation. **h** Schematic diagram of films as heat diffusion materials for a high-power LED lamp. **i** Temperature profiles which are plotted by infrared images

The comprehensive performances of GF@Cu with seamless heterointerface are investigated to verify its potential in practical applications. The electrical conductivity of the GF@Cu remains 1.1 × 10^6^ S m^−1^, which is higher than that (0.9 × 10^6^ S m^−1^) of the GF after 150 times LNS (Fig. S22). The increment of electrical conductivity is attributed to the combination of high carrier density of Cu and high mobility of GF [67]. Therefore, the GF@Cu with seamless heterointerface can also be used as a potential electromagnetic interference (EMI) shielding material [68–70]. Compared with the original GF, the EMI shielding effectiveness (EMI SE) of the GF@Cu is significantly improved, which increases from 65.4–70.3 to 74.1–79.0 dB in 8–12 GHz (Fig. S23), due to the enhanced electromagnetic field reflection loss from the highly conductive copper coating and electromagnetic field absorption loss from thicker film (Fig. S24). In terms of mechanical properties, the structure of the GF has been damaged after LNS, and its tensile strength and elongation at breakage both decrease sharply (Fig. 5a). However, the tensile strength and elongation of GF@Cu are well maintained, contributed by the repair of surface defects and the effective composite with the reinforcement phase (Cu). The tensile strength of GF@Cu is 49 MPa, ~ 9 times higher than that (5 MPa) of GF after LNS. Besides, the resistance and the coating bond of GF@Cu barely changes after 1000 bending-releasing cycles, suggesting a high tolerance for bending deformation (Figs. 5d and S17, S25). The GF@Cu demonstrates remarkable flexibility, possessing the ability to maintain structural integrity even after undergoing harsh deformation, such as repetitive bending (Fig. 5d), twisting, and complex folding of a paper crane, without incurring any breaks or fractures (Fig. 5e). Furthermore, aided by inherent flexibility of GF@Cu, the stretchable cooling components can be designed through kirigami to meet future special-shaped heat dissipation requirements (Fig. 5b). Meanwhile, the highly thermally conductive and ultra-stable GF@Cu can achieve effective heat dissipation of liquid nitrogen as an extreme cold source, inspiring new technologies for thermal management. It effectively makes temperature of heat source drop by 21 °C in two minutes (Fig. 5c). By introducing a seamless heterointerface, the GF@Cu shows a substantial improvement in thermal, mechanical, electrical performances, and stability (Fig. 5f and Table S2). Furthermore, we use different films as heat diffusion materials for a high-power LED lamp, which could verify the performance stability of modified Cu@GF than pristine GF for effective operation in extremely cold sources (Figs. 5g, h and S26). As displayed in Fig. 5g–i, the LED lamp equipped with the GF@Cu as a thermal spreader exhibits a similar heat-spot temperature with pure GF after 180 s of work, and the LED lamp equipped with the GF@Cu after LNS as a thermal spreader exhibits lower heat-spot temperature of about 0.8 °C than pure GF after LNS after 180 s. These results indicate the GF@Cu not only possesses a higher thermal conductivity but also relieves the possible urgent thermal management demands in a hostile environment.

## Conclusion

In summary, we report the undetected structural instability phenomenon of GF in response to cyclic LNS and reveal the structural failure mechanism, which follows the “permeation-diffusion-deformation” bubbling process of N_2_ molecules in the crevices and inner voids of GF. To avoid this failure, we propose a novel metal-nano armor strategy involving constructing a copper-modified graphitic film (GF@Cu) with seamless heterointerface. This well-designed interface provides superior structural stability of GF@Cu after even hundreds of LNS from 77 to 300 K. The GF@Cu maintains high thermal conductivity up to 1088 W m^−1^ K^−1^, with a degradation of less than 5%, significantly superior to that of pure GF (~ 50% degradation). Additionally, the GF@Cu exhibits excellent electrical conductivity (≈1.1 × 10^6^ S m^−1^), enhanced EMI SE performance and more stable mechanical performance, making it more suitable for extreme thermal management in complex environments. Our study overcomes the intrinsic structural instability of highly thermally conductive GF in extreme liquid nitrogen environments. Moreover, this research provides inspiration for the development of new materials for future applications and technologies involving extreme liquid nitrogen environments.

## Supplementary Information

Below is the link to the electronic supplementary material.

Supplementary file1 (PDF 2022 kb)

Supplementary file2 (MP4 13,335 kb)
