# Supplementary material for: Highly Thermally Conductive and Structurally Ultra-Stable Graphitic Films with Seamless Heterointerfaces for Extreme Thermal Management
Source: Nanomicro Lett. 2023 Dec 19;16:58. doi: 10.1007/s40820-023-01277-1 (PMC10730789; doi:10.1007/s40820-023-01277-1)
Supplement: Supplementary file 1 — Supplementary file1 (PDF 2022 kb) [file 40820_2023_1277_MOESM1_ESM.pdf]

Supporting Information for

Highly Thermally Conductive and Structurally Ultra-Stable Graphitic Films with Seamless Heterointerfaces for Extreme Thermal Management

Peijuan Zhang[1], Yuanyuan Hao[1], Hang Shi[1], Jiahao Lu[1], Yingjun Liu[1,2,*], Xin Ming[1,*], Ya Wang[1], Wenzhang Fang[1], Yuxing Xia[1], Yance Chen[1], Peng Li[1], Ziqiu Wang[1], Qingyun Su[3], Weidong Lv[4], Ji Zhou[4], Ying Zhang[5], Haiwen Lai[6], Weiwei Gao[1,2], Zhen Xu[1,2,*], Chao Gao[1,2,*]

[1]MOE Key Laboratory of Macromolecular Synthesis and Functionalization, Department of Polymer Science and Engineering, Key Laboratory of Adsorption and Separation Materials & Technologies of Zhejiang Province, Zhejiang University, 38 Zheda Road, Hangzhou 310027, P. R. China

[2]Shanxi-Zheda Institute of Advanced Materials and Chemical Engineering, Taiyuan 030032, P. R. China

[3]Beijing Spacecrafts Manufacturing Co., Ltd, Haidian District, Beijing Friendship Road 104, Beijing 100094, P. R. China

[4]Beijing Institute of Space Mechanics & Electricity, Haidian District, Beijing Friendship Road 104, Beijing 100094, P. R. China

[5]China Academy of Aerospace Aerodynamics, Beijing 100074, P. R. China

[6]Hangzhou Gaoxi Technol Co., Ltd, Hangzhou 311113, P. R. China

*Corresponding authors. E-mail: yingjunliu@zju.edu.cn (Y.J. L.); xin_ming@zju.edu.cn (X. M.); zhenxu@zju.edu.cn (Z. X.); chaogao@zju.edu.cn (C. G.)

S1 Experimental Section

S1.1 Supporting Simulation Method of Molecular Dynamics

In this work, confined case (graphene-N_2) was built for molecular dynamic simulations. Case graphene-N_2 contains 2 parallel graphene sheets (5 nm × 10 nm, slit width 0.7 nm) and 2200 N_2 molecules.

The simulation can be divided into the following steps.

Step one: The case was allowed to equilibrate for 1 ns with a time step of 1 fs by using canonical ensemble (NPT) simulations. Under environment at 77 K and 1 bar pressure. The graphene sheets were set as rigid to ensure that the atoms of the substrates were fixed during this step simulation.

Step two: After step one, the graphene sheets were set as flexible to ensure that the graphene slit was deformable during this step simulation. The case was allowed to equilibrate

for 1 ns with a time step of 1 fs by using canonical ensemble (NPT) simulations. Under environment at 77 K and 1 bar pressure. Then, another 100 ps of simulation was performed for MSD analysis, and another 1 ns of simulation was performed for carbon position analysis. The coordinates were recorded every 0.1 ps.

Step three: Following step two, the case was allowed to equilibrate for 1 ns with a time step of 1 fs by using NPT simulations. Under environment at 300 K and 1 bar pressure. And another 1 ns of simulation was performed for graphene position analysis. The coordinates were recorded every 0.1 ps.

Step four: Following step three, the N_2 were deleted, and the case was allowed to equilibrate for 1 ns with a time step of 1 fs by using NPT simulations. Under environment at 300 K and 1 bar pressure. Another 1 ns of simulation was performed for graphene position analysis. The coordinates were recorded every 0.1 ps.

In the N_2 molecular statistics section, confined case (graphene-N_2) was built for molecular dynamic simulations. Case graphene-N_2 contains 2 parallel graphene sheets (3.8 nm × 3.8 nm, slit width 0.7 nm) and 200 N_2 molecules.

The case was allowed to equilibrate for 1 ns with a time step of 1 fs by using NPT simulations, the pressure was applied in x direction. Under environment at 77 K and 1 bar pressure. The graphene sheets were set as rigid to ensure that the atoms of the substrates were fixed during this step simulation. The number of N_2 molecules between the graphene slits region with time was analyzed under 77 K and 300 K. The region between slits is (-1.6~1.6, -1.6~1.6, -0.35~0.35) nm in x, y, z.

The airebo model [S1] was used for graphene. N_2 model was taken from Vekeman et al [S2]. The van der Waals (vdW) interaction parameters of N_2 and graphene was taken from Zhang et al [S3]. All the cases were placed in periodic orthogonal boxes. And all the molecular dynamic simulations were performed by using LAMMPS software package [S4].

Furthermore, two confined cases (system 1-2) were built for Molecular Dynamic simulations to verify the enhancement mechanism of the seamless heterogeneous interface (Cu layer).

System 1 contains 2200 N_2, Cu@graphene composed slit. System 2 contains 2200 N_2, 1 Cu@graphene composed slit.

The initial configuration systems were constructed through the software of PACKMOL[1], all the N_2 molecules were randomly inserted in a cubic simulation box.

The airebo mode [S1] was used for graphene. N_2 model was taken from Vekeman et al [S2]. The van der Waals (vdW) interaction parameters of N_2 and graphene were taken from Zhang et al [S3]. The FCC model [S5] was used for Cu. The arithmetic mix rule was applied for Cu and graphene, Cu and N_2 .

The system 1 was allowed to equilibrate for 400 ps with a time step of 1 fs by using canonical ensemble (NPT) simulations, under an environment at 77 K and 101.325 kPa pressure. The slit in system 2 was set as freeze to ensure that the atoms of the substrates were fixed during

the simulation. Then, another 1000 ps of NPT simulation was performed for system 2, under an environment at 300 K and 101.325 kPa pressure. The slits in system 2 were set as unfreeze to ensure that the atoms of the substrates were flexible during the simulation.

The temperature and pressure are kept via the Nose-Hoover thermostat and Parrinello-Rahman barostat, respectively.

All the cases were placed in periodic orthogonal boxes. And all the Molecular Dynamic simulations were performed by using LAMMPS software package [S4].

S1.2 Calculation of Diffusion Coefficients from Molecular Dynamics Simulations

The diffusion coefficient can be obtained from the well-known Einstein relation, by determining the slope of the mean square displacement (MSD) over time. The MSD is given by the following equation:

$$<r^2> = <\left(r(t)\text{-}r(0)\right)^2>$$

Where $<r^2>$ is the MSD, r(t) is the position vector of the penetrant molecule at time t. If the simulation time is long enough, the diffusion coefficient is described by the following equation:

$$D = \frac{1}{6}\lim_{t\to\infty} < \frac{d}{dt}\left(r(t)\text{-}r(0)\right)^2 >$$

Here, D is the self-diffusion coefficient, t is the time, r(t) is the position vector of the penetrant molecule at time t and the angle brackets give the ensemble average.

S1.3 Supporting Simulation Method of First-principles

The first-principles calculations are carried out using the density functional theory (DFT) approach implemented in the Vienna Ab Initio Package (VASP) within the generalized gradient approximation (GGA) using the Perdew-Burke-Ernzerhof (PBE) formulation[S5-S7]. We have chosen the projected augmented wave (PAW) potentials [S8,S9] to describe the ionic cores and take valence electrons into account using a plane wave basis set with a kinetic energy cutoff of 520 eV. Partial occupancies of the Kohn−Sham orbitals were allowed using the Gaussian smearing method and a width of 0.2 eV. The electronic energy was considered self-consistent when the energy change was smaller than 10^{-5} eV. A geometry optimization was considered convergent when the energy change was smaller than 0.02 eV $Å^{-1}$. The vacuum spacing in a direction perpendicular to the plane of the structure is 18 Å. The weak interaction was described by DFT+D3 method using empirical correction in Grimme's scheme [S10, S11].

S2 Supplementary Figures

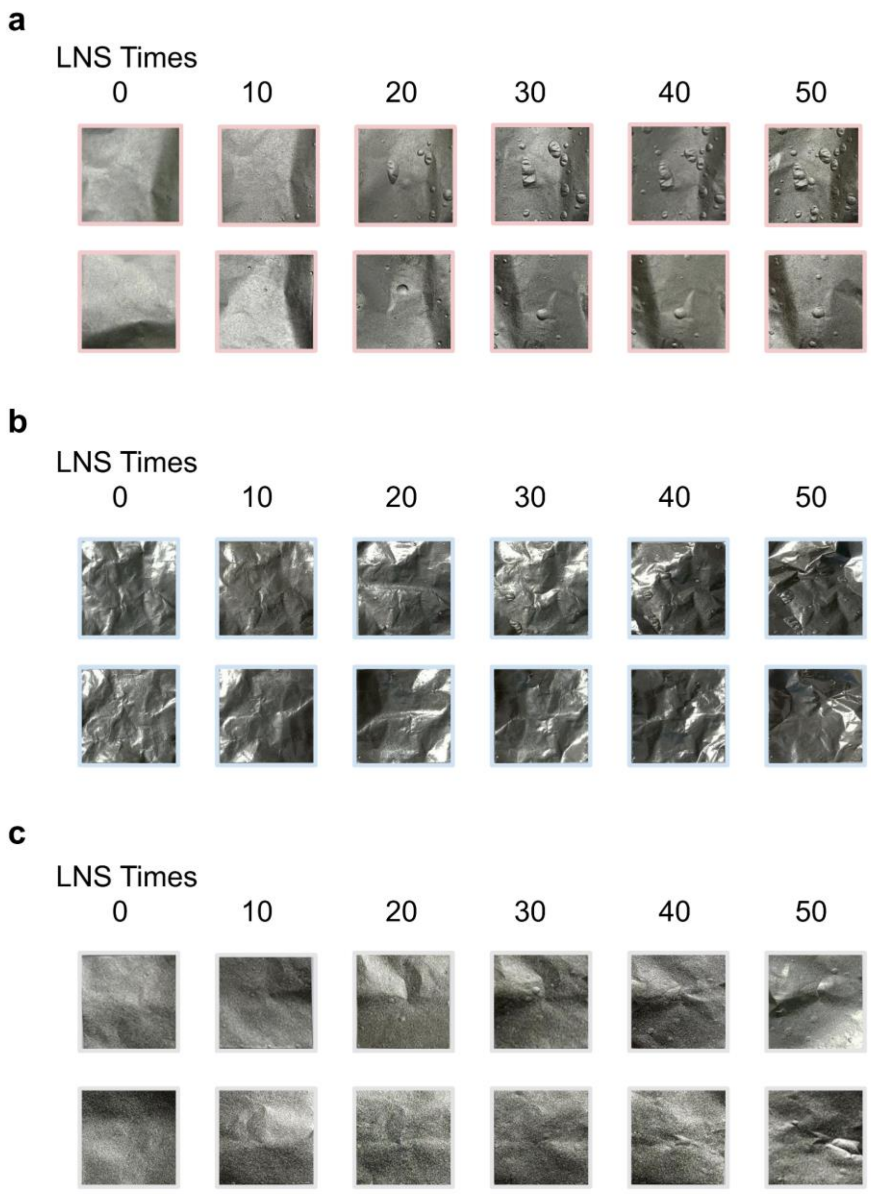

Fig. S1 Digital images of (**a**) GF-1, (**b**) GF-2 and (**c**) GF-3 surface morphologies vary with the number of LNS

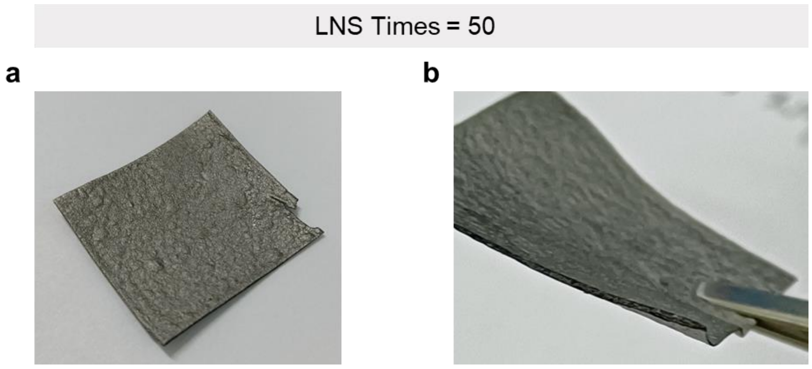

Fig. S2 Digital images of (**a**) surface and (**b**) cross-sectional morphologies of graphene film after LNS

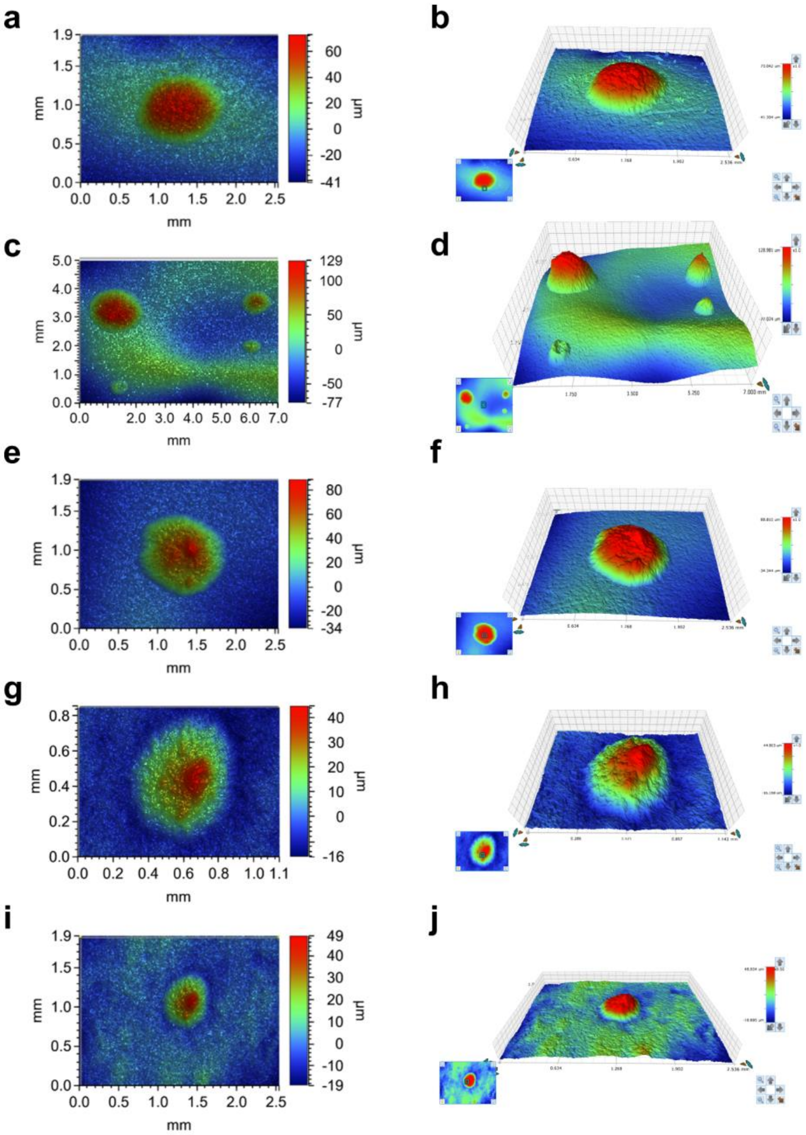

Fig. S3 Some typical stereoscopic structure images of GFs surface bubbles

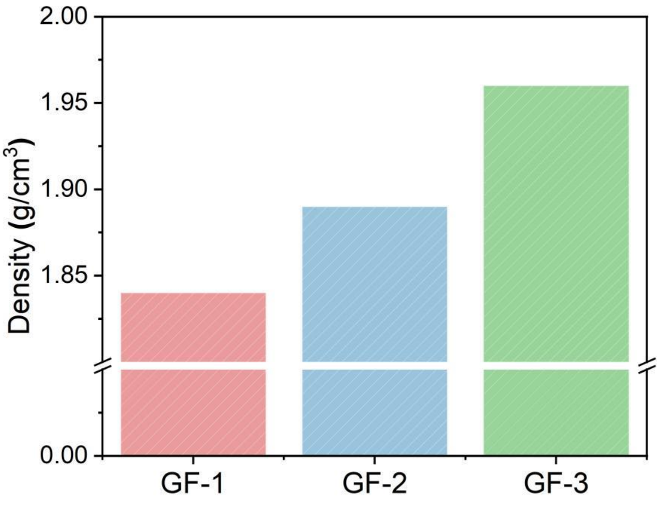

Fig. S4 Density information of three types of GFs

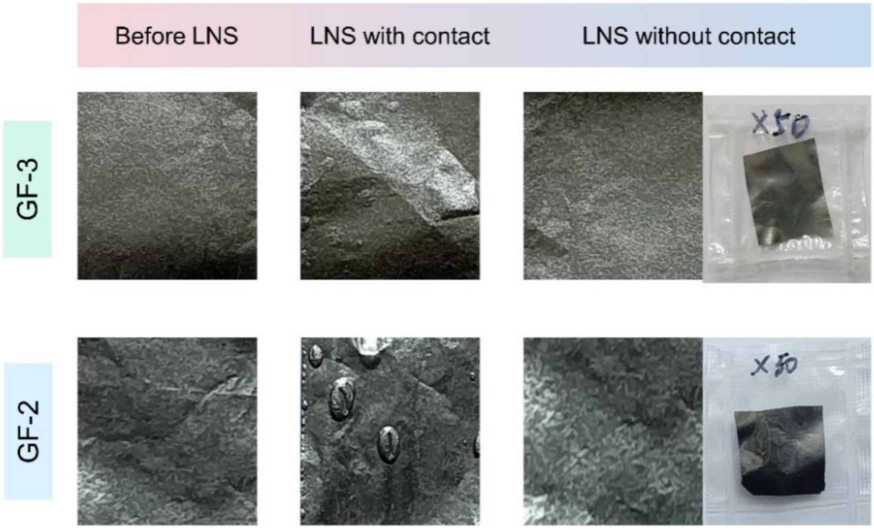

Fig. S5 LNS impacts for two GFs in different contact states with liquid nitrogen. Among them, LNS impacts without direct contacting with liquid nitrogen use plastic sealing to isolate N_2

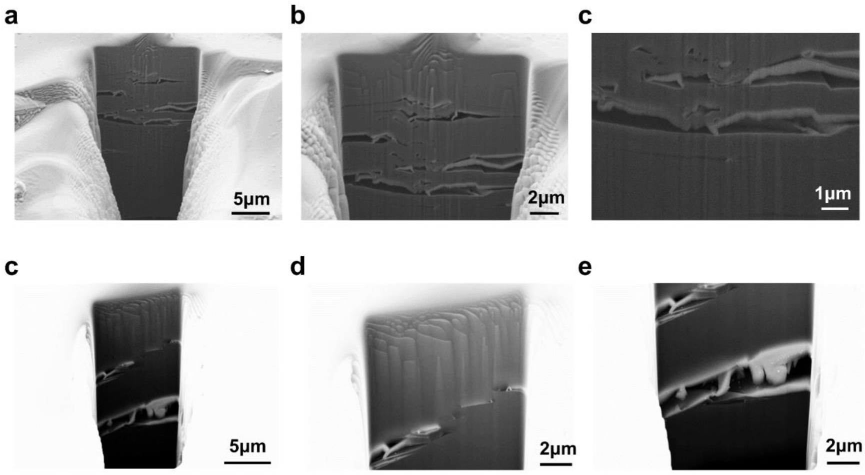

Fig. S6 SEM images of internal structure of various GFs, obtained by focused ion beam

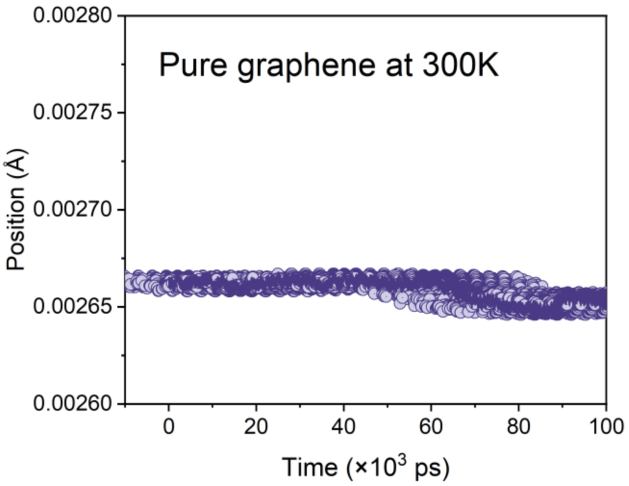

Fig. S7 The average position of the graphene sheet without a nitrogen environment from MD simulations at 300 K. The position in center of two graphene sheets slit is defined as 0

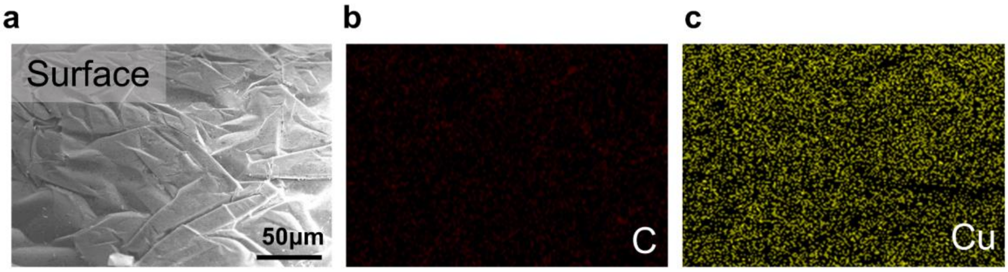

Fig. S8 SEM image and EDX mapping of GF@Cu surface

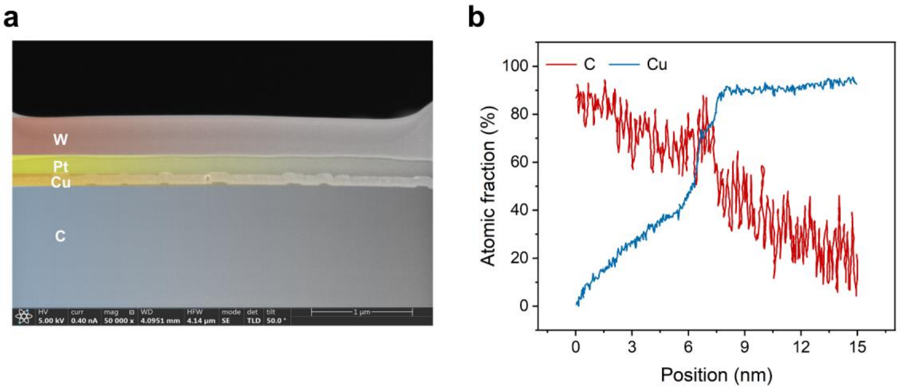

Fig. S9 (**a**) SEM image and (**b**) element distribution of Cu/C interface

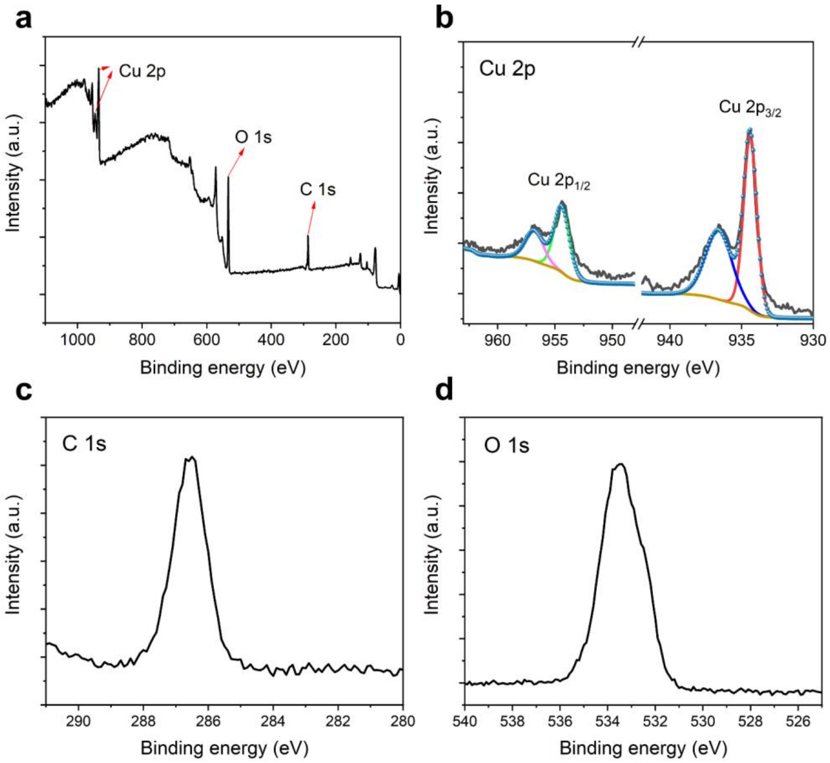

Fig. S10 X-ray photoelectron spectroscopy (XPS) spectra of GF@Cu, the clear Cu 2p peaks indicate the existence of Cu

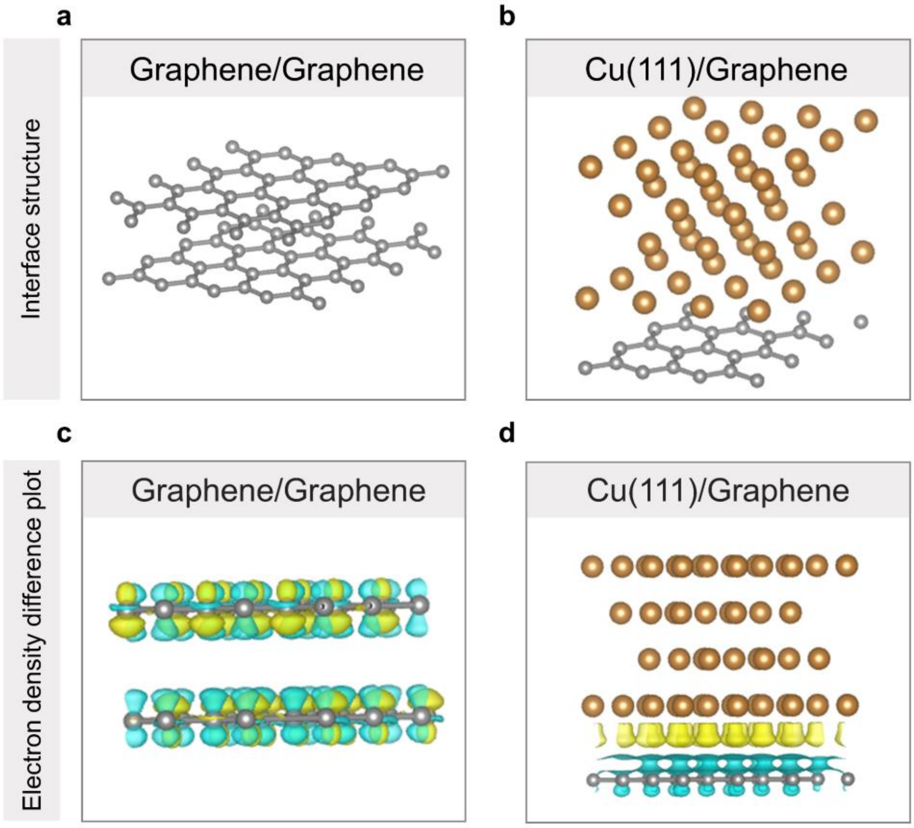

Fig. S11 Typical configuration structure of interface models: (**a**) Graphene/ graphene and (**b**) Cu(111)/graphene interface. The electron density difference plot of the (**c**) Graphene/graphene and (**d**) Cu(111)/graphene interface

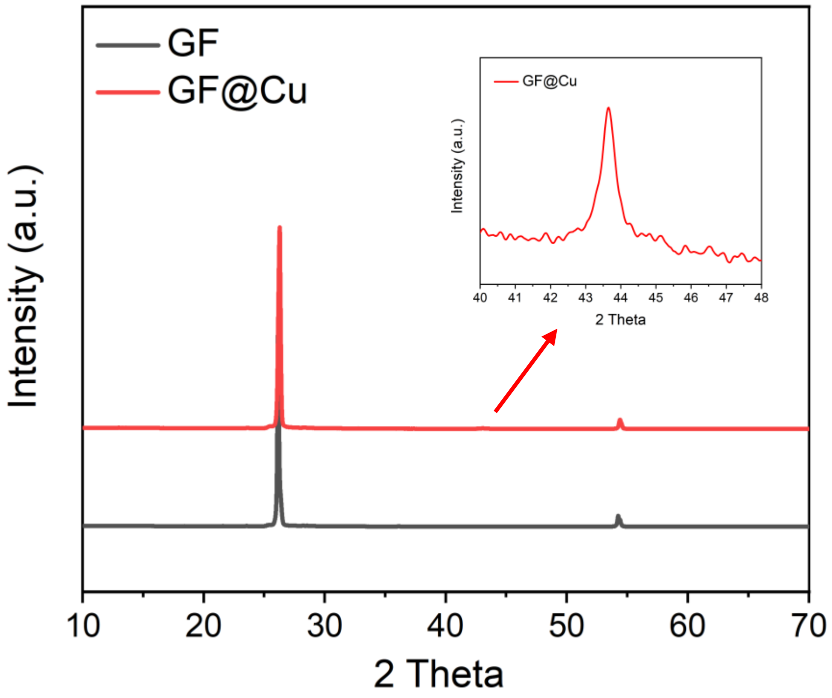

Fig. S12 X-ray diffraction (XRD) spectra of GF@Cu, indicating the retention of crystal structure and the existence of Cu

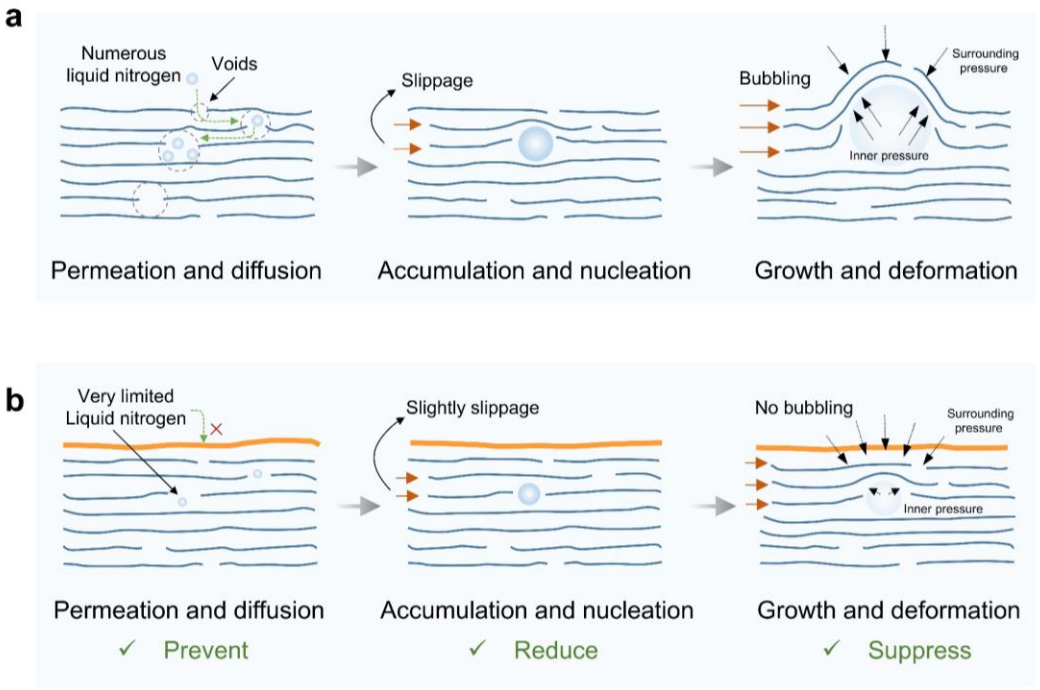

Fig. S13 (**a**) Schematic diagram of the bubbling process of original GF. (**b**) Schematic diagram of Cu@GF with seamless heterointerface, which could prevent the bubbling phenomenon

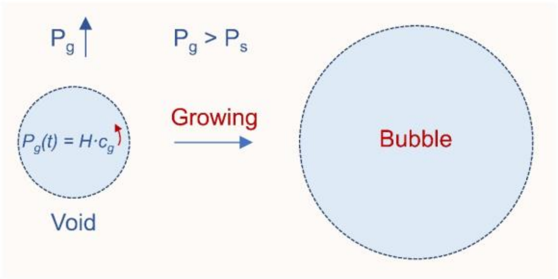

$P_g(t)$: The gas pressure at time

H: Henry constant

C_g: The concentration of gas

P_s: Surrounding pressure under graphene sheets

Fig. S14 Schematic diagram of bubble growth from Henry theory

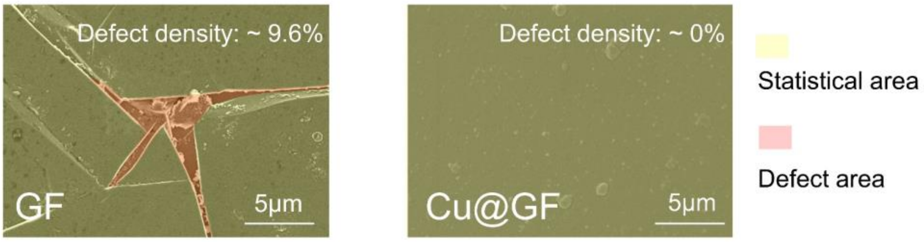

Defect density = Defect area / Statistical area

Fig. S15 The defect density of GF and Cu@GF

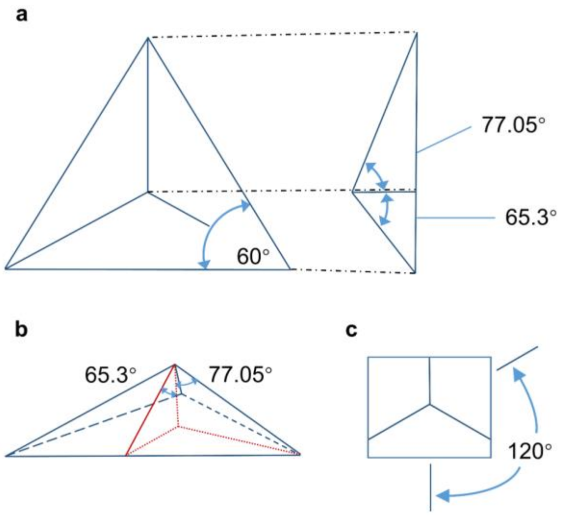

Fig. S16 Shape parameters of Berkovich indenter

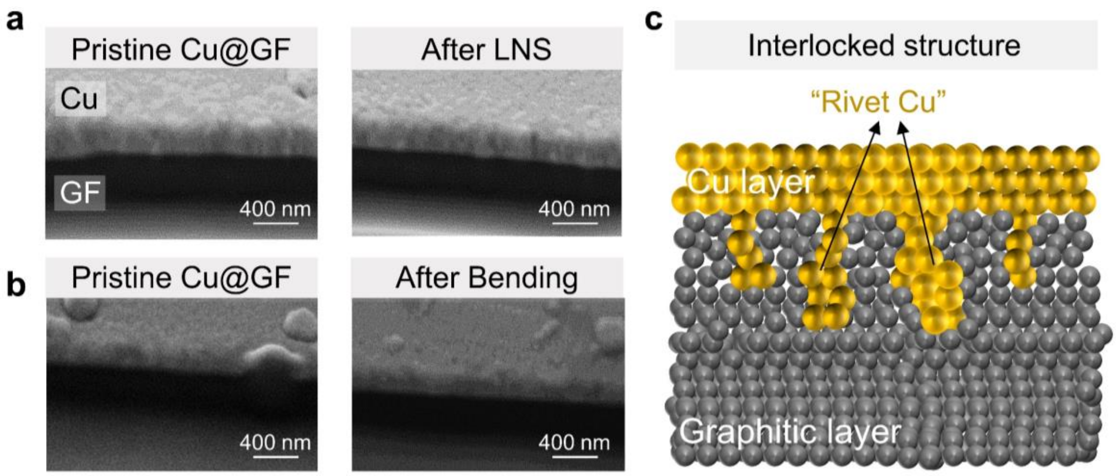

Fig. S17 (**a**) SEM images of C/Cu interface inside Cu@GF after cyclical LNS and (**b**) multiple bending. (**c**) Schematic diagram of the interlocked structure between the seamless heterointerfaces and the GF substrate, which contributes to the stability of interface bonding

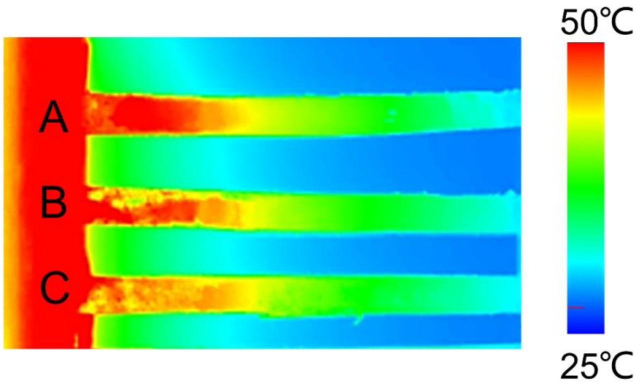

Fig. S18 Infrared image of GF with different LNS times, A = 50 times, B = 100 times, C=150 times, indicating the failure of the heat transfer path

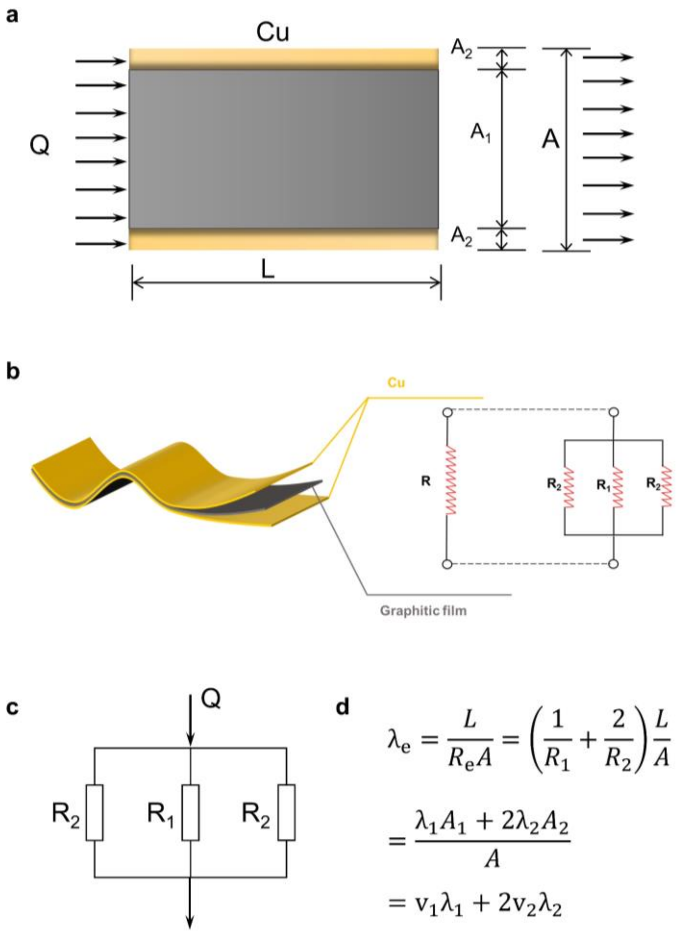

Fig. S19 (**a-c**)Schematic diagrams of composite parallel model and (**d**) calculation method of in-plane thermal conductivity by composite parallel model

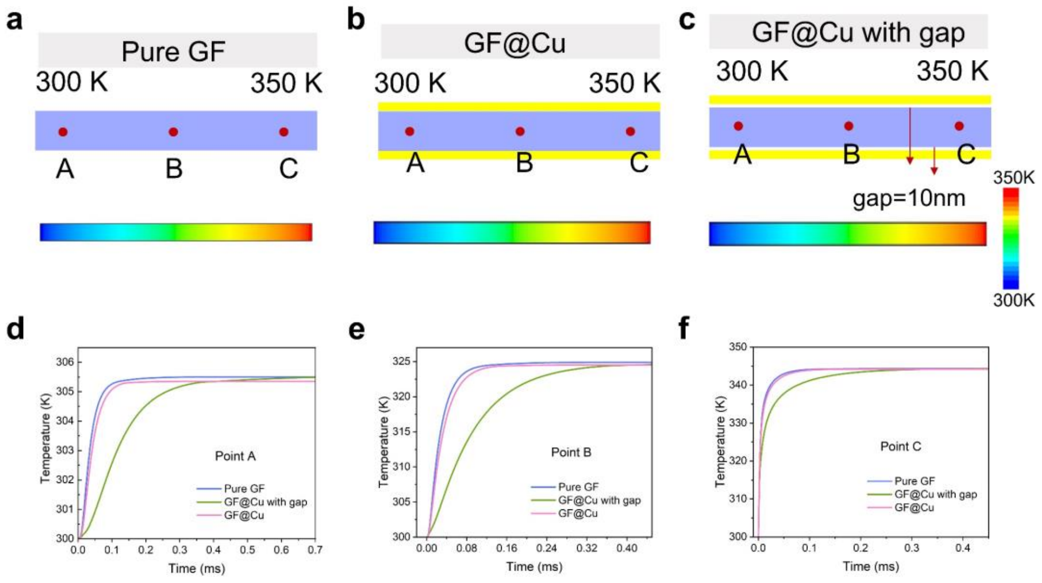

Fig. S20 Finite element analysis on the thermal conductivity, showing the heat flux transport within (**a**) pure GF , (**b**) GF@Cu and (**c**) GF@Cu with interface resistance respectively. (**d-f**) The curves are the temperature change with time of different positions in three kinds of films

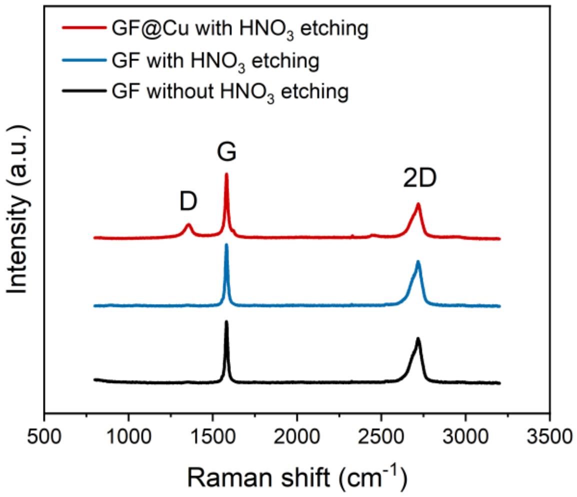

Fig. S21 Raman spectra of e-GF@Cu, e-GF and original GF, the e-GF@Cu and e-GF were etched by HNO_3

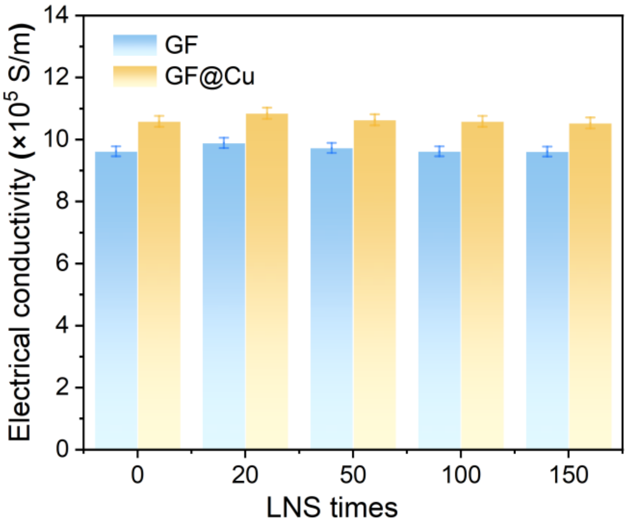

Fig. S22 Electrical conductivity of GF and GF@Cu with different LNS times

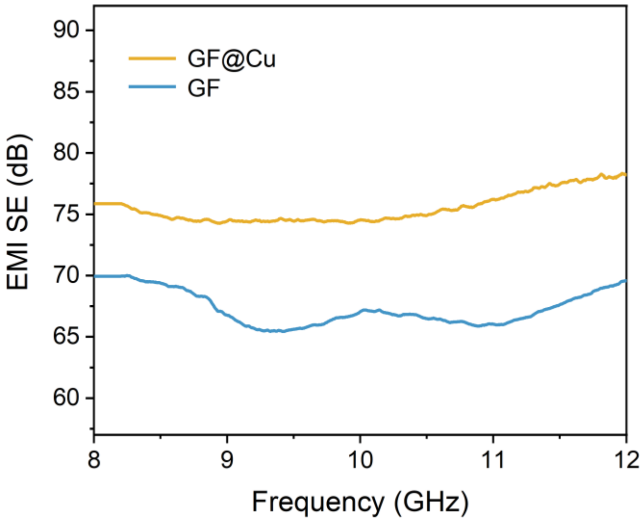

Fig. S23 The electromagnetic interference shielding effectiveness (EMI SE) of GF and GF@Cu

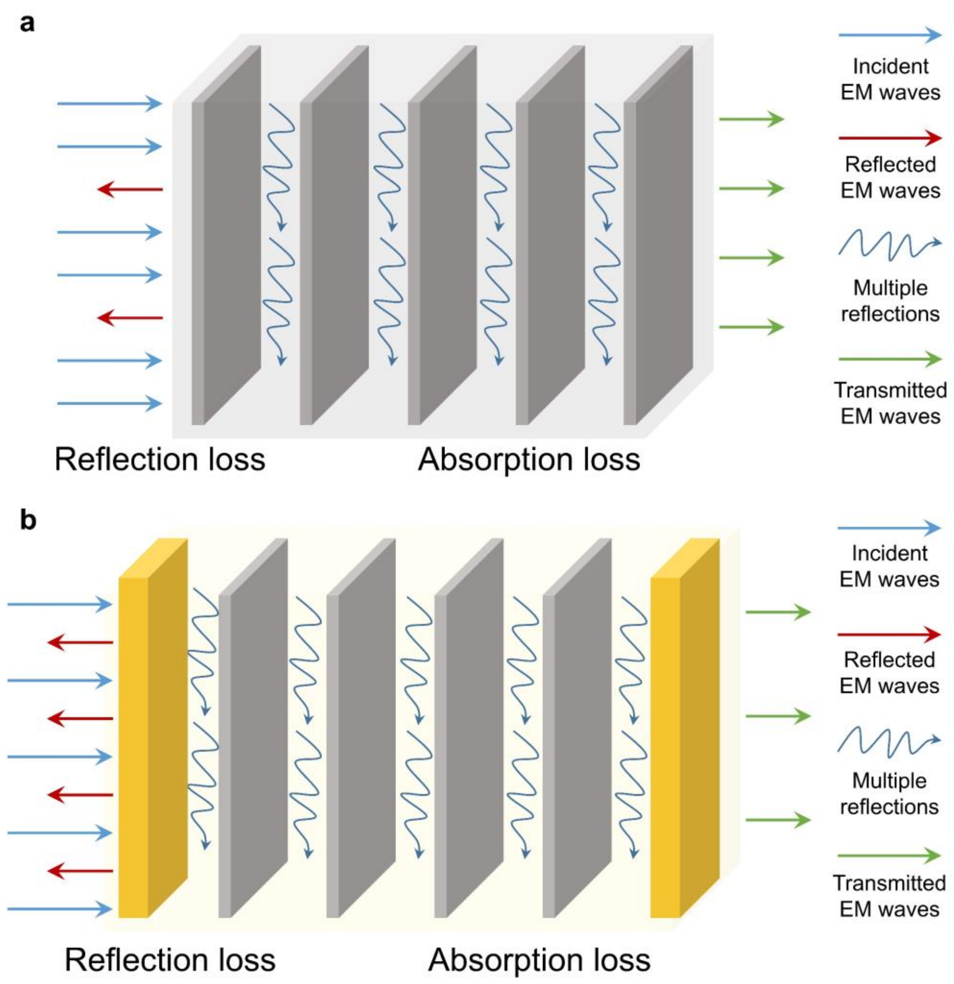

Fig. S24 Schematic of the EMI shielding mechanism of (**a**) GF and (**b**) Cu@GF

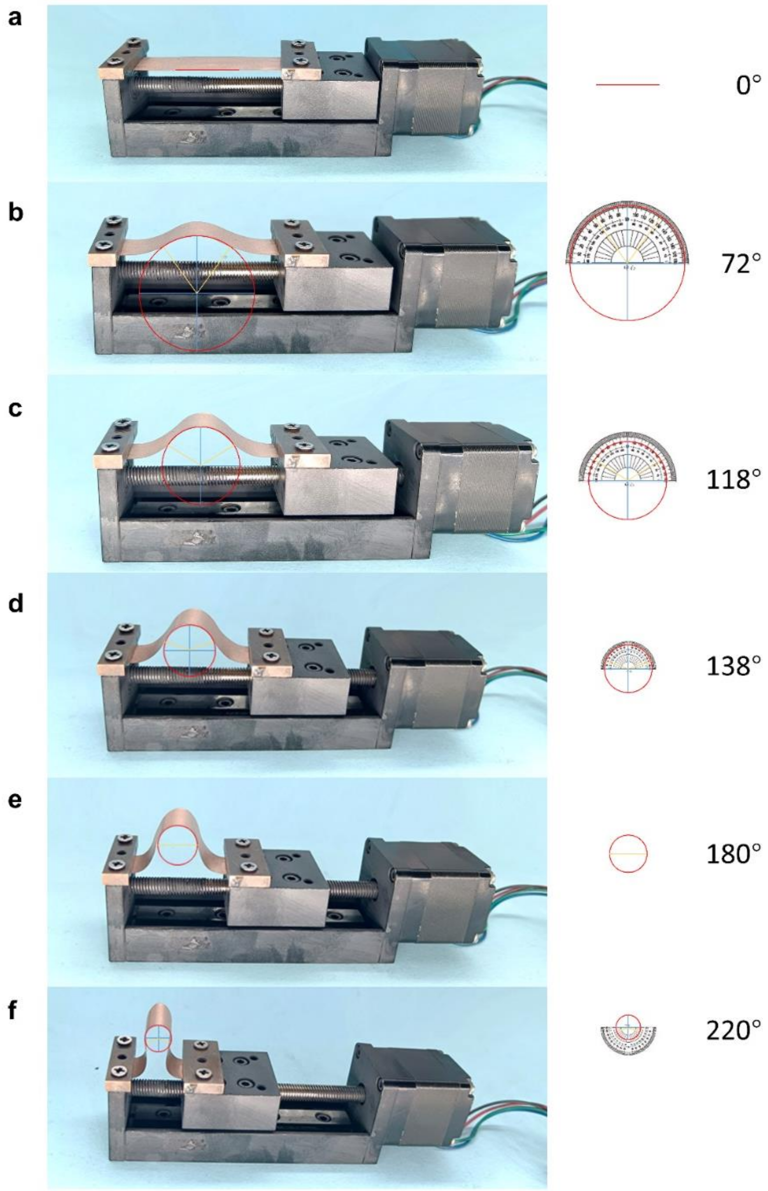

Fig. S25 Digital images of GF@Cu under different bending curvatures

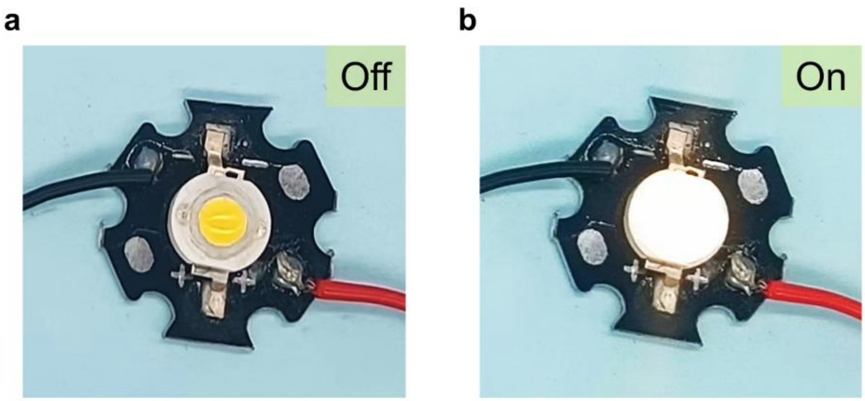

Fig. S26 Two states of the high-power LED lamp

S3 Supplementary Tables

Table S1 Nanoindentation information of GF and GF@Cu

**Types**	**Pit area (μm^2)**	**Hardness (GPa)**	**E_{eff} (GPa)**
GF	1.90	0.24	4.8
GF@Cu	1.62	0.59	8.3

Table S2 Overall performances of some reported graphitic films with this work

**Materials**	**Sample**	**Fabrication method**	**Thickness (μm)**	**Thermal conductivity (W/mK)**	**Electrical conductivity ($\times 10^5$ S/m)**	**Tensile strength (MPa)**	**EMI SE (dB)**	**Stability**	**Refs.**
This work	GF-1	A doctor blade technique and thermal reduction	25	1258	9.62	29.4	65.4-70.3	×	/
	GF-2	A doctor blade technique and thermal reduction	25	1312	9.86	31.3	64.8-71.6	×	/
	GF-2-LNS	A doctor blade technique and thermal reduction	25	728	9.83	4.5	65.1-71.8	×	
	GF-3	A doctor blade technique and thermal reduction	17	1449	10.26	32.7	66.3-73.2	×	/
	Cu@GF	Magnetron sputtering	25	1137	10.85	49.2	74.1-79.0	✓	/
	Cu@GF-LNS	Magnetron sputtering	25	1088	10.84	49.2	74.1-79.0	✓	
RGO	dfGF	A doctor blade technique and thermal reduction	20	1940	10.60	60	/	-	[S12]
RGO	annealed GP	Electro-spray deposition and thermal reduction	25	1434	1.83	58.2	/	-	[S13]
RGO	FAC-Graphene Paper	Vacuum fltration and thermal reduction	30	1529	2.23	/	/	-	[S14]
RGO	rG-O_15-3000C_PH	A doctor blade technique, thermal reduction and press	10	2025	30.3	80	/	-	[S15]

RGO	rLGO	Vacuum fltration and chemical reduction	7.5	1390	0.24	77.7	~20	-	[S16]
Polyimide	G0.25	Polyimide graphitization	35	1781	16.3	/	/	-	[S17]
Polyimide	3.5 wt.%	Polyimide graphitization	/	1092	/	/	/	-	[S18]
CNT	HADF	In situ alignment and further stretching-pressing	9.5	700.15	4.87	2950	/	-	[S19]
Composite	Graphite film/Cu/Al	PVD and vacuum hot pressing	169.5	805	/	/	/	-	[S20]
Composite	SiC@GFs/CFs (CF =Cu film)	Vacuum hot pressing	/	485.2	/	/	/	-	[S21]
Composite	GFs(Cu)/Cu	Ultrasonic-assisted electroless plating and vacuum hot-pressing sintering	/	1177.8	/	/	/	-	[S22]

Supplementary References

[S1] S.J. Stuart, A.B. Tutein, J.A. Harrison, A reactive potential for hydrocarbons with intermolecular interactions. J. Chem. Phys. **112**(14), 6472-6486 (2000). https://doi.org/10.1063/1.481208

[S2] J. Vekeman, N. Faginas-Lago, A. Lombardi, A. Sanchez de Meras, I. Garcia Cuesta et al., Molecular dynamics of CH(4)/N(2) mixtures on a flexible graphene layer: Adsorption and selectivity case study. Front Chem. **7**, 386 (2019). https://doi.org/10.3389/fchem.2019.00386

[S3] X. Zhang, H. Zhang, S. Cao, N. Zhang, B. Jin et al., Construction of position-controllable graphene bubbles in liquid nitrogen with assistance of low-power laser. ACS Appl. Mater. Interfaces **12**(50), 56260-56268 (2020). https://doi.org/10.1021/acsami.0c14857

[S4] S. Plimpton, Fast parallel algorithms for short-range molecular dynamics. J. Comput. Phys. **117**(1), 1-19 (1995). https://doi.org/10.1006/jcph.1995.1039

[S5] H. Heinz, R.A. Vaia, B.L. Farmer, R.R. Naik, Accurate simulation of surfaces and interfaces of face-centered cubic metals using 12−6 and 9−6 lennard-jones potentials. J. Phys. Chem. C **112(44)**, 17281 (2008). https://doi.org/10.1021/jp801931d

[S6] G. Kresse, J. Furthmüller, Efficiency of ab-initio total energy calculations for metals and semiconductors using a plane-wave basis set. J. Comput. Mater. Sci. **6**, 15−50 (1996). https://doi.org/10.1016/0927-0256(96)00008-0

[S7] G. Kresse, J. Furthmüller, Efficient iterative schemes for ab initio total-energy calculations using a plane-wave basis set. J. Phys. Rev. B **54**, 11169−11186 (1996). https://doi.org/10.1103/PhysRevB.54.11169

[S8] J.P. Perdew, K. Burke, M. Ernzerhof, Generalized gradient approximation made simple. Phys. Rev. Lett. **77**, 3865−3868 (1996). https://doi.org/10.1103/PhysRevLett.77.3865

[S9] G. Kresse, D. Joubert, From ultrasoft pseudopotentials to the projector augmented-wave method. Phys. Rev. B **59**, 1758-1775 (1999). https://doi.org/10.1103/PhysRevB.59.1758

[S10] P.E. Blöchl, Projector augmented-wave method. Phys. Rev. B **50**, 17953-17979 (1994). https://doi.org/10.1103/PhysRevB.50.17953

[S11] S. Grimme, J. Antony, S. Ehrlich, S. Krieg, A consistent and accurate ab initio parametrization of density functional dispersion correction (DFT-D) for the 94 elements H-Pu. J. Chem. Phys. **132**, 154104 (2010). https://doi.org/10.1063/1.3382344

[S12] S. Grimme, S. Ehrlich, L. Goerigk, Effect of the damping function in dispersion corrected density functional theory. J. Comp. Chem. **32**, 1456 (2011). https://doi.org/10.1002/jcc.21759

[S13] L. Peng, Z. Xu, Z. Liu, Y. Guo, P. Li et al., Ultrahigh thermal conductive yet superflexible graphene films. Adv. Mater. **29**(27), 1700589 (2017). https://doi.org/10.1002/adma.201700589

[S14] G.Q. Xin, H.T. Sun, T. Hu, H.R. Fard, X. Sun et al., Large-area freestanding graphene paper for superior thermal management. Adv. Mater. **26**, 4521–452 (2014). https://doi.org/10.1002/adma.201400951

[S15] C. Teng, D. Xie, J.F. Wang, Z. Yang, G.Y. Ren et al., Ultrahigh conductive graphene paper based on ball-milling exfoliated graphene. Adv. Funct. Mater. **27**, 170024 (2017). https://doi.org/10.1002/adfm.201700240

[S16] A. Akbari, B.V. Cunning, S.R. Joshi, C. Wang, D.C. Camacho-Mojica et al., Highly ordered and dense thermally conductive graphitic films from a graphene oxide/reduced graphene oxide mixture. Matter **2**, 1198–1206 (2020). https://doi.org/10.1016/j.matt.2020.02.014

[S17] P. Kumar, F. Shahzad, S. Yu, S.M. Hong, Y.H. Kim et al., Large-area reduced graphene oxide thin film with excellent thermal conductivity and electromagnetic interference shielding effectiveness. Carbon **94**, 494-500 (2015). https://doi.org/10.1016/j.carbon.2015.07.032

[S18] S. Li, Z. Zheng, S. Liu, Z. Chi, X. Chen et al., Ultrahigh thermal and electric conductive graphite films prepared by g-C3N4 catalyzed graphitization of polyimide films. Chem. Eng. J. **430**, 132530 (2022). https://doi.org/10.1016/j.cej.2021.132530

[S19] M. Weng, L.F. Jian, X. Feng, X. Luo, J. Hu et al., High oriented graphite film with high thermal conductivity prepared by pure polyimide film formed with catalyst pyridine. Ceram. Int. **47**(17), 24519–24526 (2021). https://doi.org/10.1016/j.ceramint.2021.05.170

[S20] H. Zhan, Y.W. Chen, Q.Q. Shi, Y. Zhang, R.W. Mo et al., Highly aligned and densified carbon nanotube films with superior thermal conductivity and mechanical strength. Carbon **186**, 205–214 (2022). https://doi.org/10.1016/j.carbon.2021.09.069

[S21] Y. Huang, Y. Su, X. Guo, Q. Guo, Q. Ouyang et al., Fabrication and thermal conductivity of copper coated graphite film/aluminum composites for effective thermal management. J. Alloys Compd. **711**, 22-30 (2017). https://doi.org/10.1016/j.jallcom.2017.03.233

[S22] X. Han, Y. Huang, J. Wang, S. Zhang, Z. Ding et al., Fabrication of graphite films/copper composites by vacuum hot pressing for high-efficiency thermal management property. Compos. Commun. **24**, 100665 (2021). https://doi.org/10.1016/j.coco.2021.100665

[S23] X. Wang, Y. Su, Q. Ouyang, C. Zhu, H. Cao et al., Fabrication, mechanical and thermal properties of copper coated graphite films reinforced copper matrix laminated composites via ultrasonic-assisted electroless plating and vacuum hot-pressing sintering. Mater. Sci. Eng. A **824**, 141768 (2021). https://doi.org/10.1016/j.msea.2021.141768
